# Entrofy your cohort: A transparent method for diverse cohort selection

**DOI:** 10.1371/journal.pone.0231939

**Published:** 2020-07-27

**Authors:** Daniela Huppenkothen, Brian McFee, Laura Norén

**Affiliations:** 1 Department of Astronomy, DIRAC Institute, University of Washington, Seattle, WA, United States of America; 2 The Washington Research Foundation Data Science Studio, The University of Washington eScience Institute, University of Washington, Seattle, WA, United States of America; 3 Center for Data Science, New York University, New York, NY, United States of America; 4 Music and Audio Research Lab, New York University, New York, NY, United States of America; 5 Obsidian Security, Newport Beach, CA, United States of America; Indiana University Bloomington, UNITED STATES

## Abstract

Selecting a cohort from a set of candidates is a common task within and beyond academia. Admitting students, awarding grants, and choosing speakers for a conference are situations where human biases may affect the selection of any particular candidate, and, thereby the composition of the final cohort. In this paper, we propose a new algorithm, *entrofy*, designed to be part of a human-in-the-loop decision making strategy aimed at making cohort selection as just, transparent, and accountable as possible. We suggest embedding *entrofy* in a two-step selection procedure. During a merit review, the committee selects all applicants, submissions, or other entities that meet their merit-based criteria. This often yields a cohort larger than the admissible number. In the second stage, the target cohort can be chosen from this meritorious pool via a new algorithm and software tool called *entrofy*. *entrofy* optimizes differences across an assignable set of categories selected by the human committee. Criteria could include academic discipline, home country, experience with certain technologies, or other quantifiable characteristics. The *entrofy* algorithm then yields the approximation of pre-defined target proportions for each category by solving the tie-breaking problem with provable performance guarantees. We show how *entrofy* selects cohorts according to pre-determined characteristics in simulated sets of applications and demonstrate its use in a case study of Astro Hack Week. This two stage candidate and cohort selection process allows human judgment and debate to guide the assessment of candidates’ merit in step 1. Then the human committee defines relevant diversity criteria which will be used as computational parameters in *entrofy*. Once the parameters are defined, the set of candidates who meet the minimum threshold for merit are passed through the *entrofy* cohort selection procedure in step 2 which yields a cohort of a composition as close as possible to the computational parameters defined by the committee. This process has the benefit of separating the meritorious assessment of candidates from certain elements of their diversity and from some considerations around cohort composition. It also increases the transparency and auditability of the process, which enables, but does not guarantee, fairness. Splitting merit and diversity considerations into their own assessment stages makes it easier to explain why a given candidate was selected or rejected, though it does not eliminate the possibility of objectionable bias.

## Introduction

Selecting a cohort from among a pool of candidates is a common task within and beyond academia. Cohort selection scenarios include admitting students to degree programs, awarding fellowships and grants, selecting speakers for a seminar series, panelists for a conference, or participants for space-limited workshops. A frequent challenge in all of these cases is the inherent limitation of available slots or jobs, as well as the practical need to select the best cohort out of a much larger set of available candidates. At present, this type of selection in academia is generally performed by committees convened to judge each candidate or proposal, taking all of their personal and professional characteristics into account at once.

### Diversity in teams

There is a large body of research studying the effects of group composition on measurable outcomes across a range of different contexts, but particularly in the context of employment [1]. Diversity within collectives (also described as heterogeneity) at the broadest level refers to any compositional differences between individuals within a group. Diversity may be measured as differences in individuals’ ascribed traits (e.g. involuntary characteristics), their achieved traits [2, 3], or as a structural property of a collective [4–6]. Within these frameworks, diversity attributes are then categorized in terms of their likely effect on group-level or individual-level outcomes. Many approaches that conceptualize diversity as a personal attribute introduce typologies that capture the perceptibility of differences, i.e. how observable a given diversity attribute is [7, 8]. For example, [9] and [10] suggest a distinction between surface-level and deep-level attributes. Here, surface-level attributes describe innate differences in people that are readily apparent or easily assessed, which can include demographic categories like age, ethnicity, and gender. Deep-level attributes are acquired attributes that may be task-relevant and not easily apparent. In a similar vein, [11] distinguishes between job-related diversity (e.g. expertise) and non-job related diversity (e.g. demographic attributes). Many researchers posit that these typologies map fairly directly onto theories of intergroup relations (see, e.g. [1] for an overview). For example, there is some evidence that diversity categories commonly grouped under job-relatedness and deep-level types of diversity, such as diversity across functional backgrounds in a company, have a positive relationship with performance [12], in particular when performance measures focus on creativity and innovation rather than efficiency [13]. This fits within the diversity-cognitive resource view of diversity [14, 15]: as group members diverge in terms of knowledge and experience, groups can more effectively generate ideas and innovations. Conversely, inter-group theories such as social identity theory [16] and the similarity-attraction paradigm [17] suggest that surface-level diversity may inhibit outcomes both in terms of performance and satisfaction [18, 19], because of an increased factionalization of the group due to individuals forming ingroup/outgroup distinctions based on surface-level characteristics.

While these theories and conceptualizations provide convincing narratives, evidence in practice is equivocal and highly context-dependent [7, 8, 12, 13]. On the whole, research on observable diversity characteristics suggests that there are negative influences on process variables, in particular when efficiency is used as a performance measure [13]. Attribute dissimilarity also impacts feelings of group identification at an individual level, which in turn affect outgroup members’ self-esteem and well-being [20, 21]. At a group level, however, meta-analyses do not reliably point to differences in group cohesion for diversity attributes in the job-relatedness category [12]. In an academic context, there is evidence that cultural diversity (as a deep-level diversity attribute) in academic departments leads to higher performance and satisfaction, whereas gender diversity (as a surface-level attribute), may be negatively associated with satisfaction [22, 23]. However, these results rest on self-reported measures of performance and satisfaction, which meta-analyses have shown to produce stronger effects [12]. Hierarchical effects might also be important: [24] consider learning outcomes of (predominantly Latinx) professional trainees in terms of the demographic attributes of their trainers, as well as in terms of the overall group composition, and find that knowledge acquisition depends on the trainer’s gender for women trainees, but not men trainees. The authors suggest that this relationship, where women trainees have higher learning outcomes when taught by trainers who are men, is consistent with social identity theory, where women trainees’ gender identity is less salient, and thus helpful in building a positive self-identity. They also suggest that having a woman trainer, conversely, makes the trainee’s gender more salient and thus activates stereotype threat, inhibiting learning outcomes [25, 26]. [24] interestingly find that the gender composition of the classroom had no effect on the learning outcomes of trainees of any gender, though they noted that classroom composition may have influenced other training outcomes (such as the number of questions asked). There is evidence that individuals’ attitudes about the value and effects of diversity in teams can affect team outcomes [27]. A similarity in beliefs about diversity has been shown to have a positive effect on performance. In addition, time-dependent effects have been observed, suggesting that the effects of surface-level attributes, because they readily apparent, dominate during early stages of teamwork [9, 28]. As time goes on and individuals discover deep-level attributes about their team members, the latter produce the more salient effects. This is of particular importance in the context of short, time-bounded events such as those considered in the later part of this paper: academic workshops generally only last a few days, which may not be enough time for deep-level features to become relevant enough to mediate outcomes.

[13] suggest that the mixed outcomes of research studying links between types of diversity at individual and group outcome levels can be explained in part by simplistic conceptualizations of diversity and how they affect inter-group relations. Similarly, [1] calls for the development of an improved theoretical underpinning of diversity conceptualizations as a foundation to explain observed (null-)effects. More recent, multi-dimensional conceptualizations aim to view diversity as a group-level, compositional attribute that affects processes and outcomes. Approaches in this category include research on demographic faultlines [29] as well as gestalt conceptualizations such as the cultural mosaic perspective [30], where diversity is viewed as a composite of geographic, demographic and associative features of culture. [31] suggest a typology of three different diversity types: separation diversity, which describes differences in attitudes, beliefs and values; variety diversity, which encodes differences in experiences and knowledge; and disparity, a measure of differential access to valued resources such as wealth and access to opportunity.

Learning and collaborations in the academic sphere have undergone a period of change with the advent of new methods and formats such as active-learning techniques and hackathons [32]. This, in turn, has generated interest in how participants learn and collaborate at these venues and what role diversity does and should play. Hackathons have generally been suggested as a platform for peer learning [33], and [34] suggest that brainstorming practices in particular allow minority group members to participate more fully than they may otherwise. However, they also note that hackathon-like settings are particularly prone to stereotyping behaviour because they are short-term and intensive, with insufficient time for team members to develop a deeper understanding of the individuals in their group. In interviews, a participant described a general intimidation based on being one of few visible minority participants in the room, and difficulty participating equitably during group discussions. In contrast, [35] report that early interventions can have a positive effect on the integration of minority students on a longer timescale. In a study of students in health education, a group-work intervention was linked to a higher degree of intercultural knowledge and problem solving among both cultural majority and minority health teachers-in-training. The authors suggest that early classroom intervention may promote intercultural engagement and prevent in-group formation along cultural boundaries. Similarly, research suggests that knowledge heterogeneity promotes innovation in student teams during an innovation challenge [36], and a combination of cultural, disciplinary and educational diversity enhances both creativity and productivity in student groups in a design course [37].

Because the relationships between diversity variables and outcomes are so varied and complex, drawing conclusions for the purpose of selecting cohorts is difficult. [38] suggests a number of guidelines: tasks requiring a high degree of cooperation may be better completed by more homogeneous groups (in accordance with social identity theory), while tasks involving collective problem-solving may benefit from a diversity of knowledge and perspectives (broadly following the diversity-cognitive resources perspective). Finally, they caution that tasks involving individual problem-solving may be subject to peer effects, where diversity produces externalities in which the behaviour of one group affects the behaviour of another (for example disruptions in a classroom, [39]). They also note that a minimum representation may be crucial to mitigate the perceived tokenization of individuals.

### Cohort selection: Overview

Current processes of cohort selection are typically adjudicated by committees that consider each candidate holistically, taking all of their personal and professional characteristics into account at once. This process is unlikely to effectively consider the varied effects of different types of diversity on the outcomes due, at least in part, to cognitive overload. It is computationally difficult to consider up to 25 categories of variation for each candidate, compared simultaneously to 25 attributes for every other candidate. Additionally, the process may weakly or inconsistently consider measures of personal satisfaction, measures of group cohesion, and measures of performance that fall outside narrowly defined success metrics. In response to scarce cognitive and temporal resources, committees may fall back on mental heuristics that lead to biased outcomes. This is especially true when the cohort to be selected is large. In the context of employment, where single individuals or very small cohorts are selected from a pool that is itself not very large, it might be possible for a committee to keep all relevant information simultaneously in play, giving careful consideration to how a specific decision might impact outcomes for the team or unit. For large cohorts, however, this would require simultaneously considering the properties of the entire candidate set, the desired cohort to be selected, and each individual candidates’ personal attributes. This is a cognitive feat well outside the limits of what current research suggests human memory can achieve [40]. We argue this process may yield suboptimal results due to the conflation of professional/academic merit and a range of other personal characteristics [38] mediated by heuristics and biases. With respect to professional and academic merit, the goal is generally to choose the best possible candidates. With respect to certain types of meritorious characteristics, say, the proven ability to publish, all candidates are competing with the same criteria in mind. With respect to other characteristics—for instance, strength in certain computational tasks, or age range—it may be more advantageous to select a cohort where diversity is carefully managed and calibrated towards achieving the overarching objectives. We propose to improve on the typical selection process by introducing a computational tool designed to bear the cognitive load of assessing cohort diversity across assignable criteria. Our assumptions are that: 1) diversity across different attributes should be actively managed to account for the effects of diversity on group outcomes, and to inform interventions during team work that allow diversity to act as a mediator of satisfaction and performance; 2) merit review requires the cognitive integration of multiple sources of complex information, and may therefore be too nuanced for computational assessment; 3) meeting targets across multiple criteria of deep-level and surface-level diversity is too complex for human cognition and is subject to human bias [41]. If these assumptions hold, traditional selection committees may be unable to efficiently select optimal cohorts and any fully computational approach would also be unlikely to produce optimal cohorts. Thus, we suggest splitting the selection into a (possibly blind) merit review process and an algorithmic procedure that approximates numerical targets on diversity criteria carefully defined by each cohort selection committee. There are two caveats to be mentioned here: no procedure, no matter how quantitative, is free of bias. Because algorithms are constructs created by humans, they encode existing beliefs and biases [42]. In particular, one implicit assumption of the proposed procedure is that the assessment of merit can be separable from diversity which is assessed in a separate second step. However, as we lay out in more detail in the Discussion, diversity attributes that fall into the *disparity* category in the typology proposed by [31], i.e. attributes that encode differences in access to valued resources and privilege, may not be independent of criteria used during the merit selection stage. Disparity attributes may thus act to exclude meritorious candidates during the first step if not taken into account. While the proposed procedure cannot, in itself, remove bias from human selection processes, it aims to contribute to improving cohort selection through two specific goals: 1) assist human committees in the cognitively labour-intensive task of optimizing a cohort across multiple relevant categories of diversity simultaneously, and 2) provide a quantitative framework that allows transparency and accountability for audit and evaluation at each stage in the process.

### Candidate selection in the context of employment

Much has been written about the inherent biases in selecting candidates for employment [43–49]. In particular, despite ample evidence to the contrary [50, 51], is a prevailing opinion that successful candidate selection can be learned and intuitive judgments are predictive of future job performance. This often leads committee members to make overconfident predictions about the success of the selection procedure with respect to employee productivity, and an underestimation of the inherently stochastic processes involved [52, 53]. Additionally, humans tend to be swayed by stories over facts: in the employment context, this can lead to decisions that defy evidence and logic [54]. Other inherent biases have been found to affect selection outcomes and salaries with respect to gender [55, 56] and race [57, 58] based on the candidate’s name and/or appearance. In several replicated studies across different problems, unstructured interviews have been found to produce worse outcomes than grades, general intelligence tests, and structured interviews with clear selection criteria [49, 53], yet selection procedures continue to be dominated by panels relying largely on experience, intuition, and debate over one applicant at a time [59] rather than systematic consideration of possible cohorts of similar collective merit. Additionally, panels tend to rely more heavily on the representativeness heuristic (assuming that because something is more representative, it is also more likely [60]) and show more overconfidence in their judgments than individuals do [61]. These findings stand in contrast to the goal of most selection procedures: to choose the best set of candidates conditional on minimum requirements and goals dictated by the situation.

While there is a vast literature regarding hiring practices, there is little empirical research about academic conference selection procedures. Still, conferences are an important cornerstone of academic careers: they provide venues for learning the most recent scientific results (often ahead of publication), for presenting one’s own work, and for networking [62]. Conference presentations are considered an important measure of academic success [63] and increase research visibility. This is particularly the case for early-career researchers and those from international or lower-tier institutions [64]. Workshop attendance [65] and conference presentations [66] can boost the probability of early-stage female faculty members getting published and cited. For example, [67] find that the number of conferences attended predicts post-conference publications, presentations, and current research activity for attendees of a research methods conference for primary care practitioners. There is thus a legitimate concern that the same biases affecting hiring practices may analogously influence selection of abstracts and participants at academic workshops and conferences. For example, [68] find that for major medical conferences, an unblinded selection procedure favours candidates from the United States, from English-speaking countries and from prestigious universities.

### Diversity optimization in recommender systems and information retrieval

The problem of algorithmically selecting a diverse cohort from a candidate pool is related to diversity optimization within the contexts of information retrieval (IR) [69] and recommender systems [70], where a set of documents is ordered by relevance to a search query, and a diverse set of results is selected to minimize information redundancy between elements of the set. Recommender systems typically rank items by similarity to items in the user’s activity history, producing a ranked list of items (e.g., movies, articles, or songs) which ideally appeal to the user. Because items well-matched to the user profile are also likely to be well-matched to one another, this can lead to homogeneity in the recommendations, which in turn may frustrate users. In addition, user queries in both recommender systems and IR often come with a large amount of uncertainty in terms of user need [71]. This, along with behavioural research indicating that individuals appreciate and seek out variety [72], is a key motivation for including metrics of diversity between items in the problem formulation.

Research on recommender systems and information retrieval systems generally distinguishes between *diversity* as the differences between items in a set, and *novelty* as the difference between present and past experience. While much research concerns introducing novelty—and, as a related concept, serendipity—into recommender systems, there is no analogue for novelty in the context of cohort selection, and we do not discuss it any further except where needed.

There are a number of different proposed frameworks to introduce diversity into recommender systems, including result-diversification and re-ranking, and clustering methods. Result-diversification methods generally incorporate metrics of both similarity (or relevance) and diversity. Ranked lists of candidate items are produced according to relevance metric, and subsequently re-ranked to enhance the diversity or novelty of the items at the top of the list (e.g. [73, 74]). Many approaches use an objective that jointly optimizes relevance and diversity, using a parameter to express the trade-off between both metrics for a candidate set. This objective can be used, for example, in greedy algorithms that re-rank an initial set selected using e.g. a nearest-neighbour collaborative filtering method [75]. Some approaches avoid the explicit trade-offs between diversity and relevance included in most formulations that treat both at the same time. Perhaps the closest approach to the one taken in this paper is by [76], who maximize diversity under a relevance constraint by selecting the most diverse cohort from a candidate set where all items have equal relevance.

Methods using clustering, conversely, cluster items in a user’s profile, then pick (representative) items similar to each cluster to recommend [70]. Approaches involve clustering the candidate set and subsequently recommending representative items of each cluster [77], as well as graph-based recommendation approaches [78]. Because these methods depend on representing each query as a collection to be clustered (i.e., the user’s previously consumed items), they are generally not applicable to the problem of cohort selection where there is no obviously analogous collection interpretation.

Recommender systems assume that there is an *inherent* trade-off in the utility, quality or relevance of recommendations and the diversity of recommendations [70]. There is also often uncertainty in the different attributes exhibited by an item that are important for the similarity computation. Recommender systems therefore aim to diversify potential recommendations to increase the probability that a returned recommendation will match a user’s query. Many metrics and algorithms developed in this context include an explicit trade-off between relevance and diversity.

While some similarities between recommender systems and cohort selection exist, there are a number of important differences. At the most fundamental level, recommender systems aim to generate an ordered list of options in the hope that *at least one* will appear to a user. This is a very different objective to cohort selection, where the output is fundamentally *unordered*, the overall composition of the cohort is expected to affect outcomes on a global and individual level, and complex inter-group effects both moderate and mediate those outcomes. Recommender systems are almost always formulated in the context of consumer behaviour; research on the latter informs designs of the former. However, consumer behaviour may differ from behaviour of individuals in social groups in fundamental ways: while research shows that individuals are generally variety-seeking [72], individuals in groups tend to prefer to interact with other group members that are similar to them (similarity-attraction paradigm [17]) and tend to form subgroups according to readily apparent characteristics (social identity theory [16]). It is therefore not given that design choices in the context of consumer behaviour are appropriate in the context of cohort selection. Because there is a large amount of inherent variance in committee-generated ratings on merit categories, there exists no well-ordered ranking of merit [79] in most cohort selection processed, i.e. while we can expect that candidates in the top 30% are generally more meritorious according to the defined categories than candidates in the bottom 30%, a precise ranking is not meaningful. This is in opposition to many recommender systems, which produce well-ordered lists of candidates. It is therefore unclear that common formulations of the problem as applied to recommender systems would be meaningful. In addition, explicitly trading off merit and diversity categories in the context of cohort selection, as proposed in most versions of recommender systems, is generally unnecessary, and ethically and politically questionable at best. We assume here that for any reasonable cohort selection problem encountered where our proposed approach applies, a diverse and meritorious pool of candidates exists (e.g. [80]). While there are limits on the assumption of separability between merit selection and selection across diversity categories we make in this approach (see also section *Limitations*), the effects that diversity categories may have on merit selection are too nuanced to be encoded in a straightforward relationship as is the case in many recommender systems, and are better addressed during merit review. Because research on the effect of quotas on demographic attributes has shown that quotas, while effective, can lead to active discrimination of qualified individuals [81], we reject from consideration any algorithm that makes an explicit merit-diversity trade-off. Subjecting participants, especially those from minority backgrounds, to discrimination runs counter any reasonable cohort selection goal. Recommender systems that include mechanisms for including diversity or novelty generally do not fine-tune how recommendations are diversified, they aim to maximize diversity across all or a subset of attributes as much as possible. This, too, does not match the context of cohort selection, where maximizing diversity across all available attributes may be desirable for some categories (e.g. academic subfield), but not others (e.g. career stage, where one may wish to admit more graduate students than undergraduate students, for example).

We opt for a clear separation between determination of individual merit (achieved characteristics) and the creation of an optimally diverse cohort. Separating these two steps obviates the need for finding a consensus ranking of candidates and provides an audit trail for determining how each inclusion/exclusion decision was made. Accountability is critical for establishing trust in a system that directly impacts people [82] and may be required by equal opportunity legislation.

### Overview of this manuscript

In this paper we introduce a new algorithm as part of a larger strategy to make cohort selection in academic and non-academic contexts more accountable and transparent in order to allow committees to introspect on their decisions and processes, probe for inherent biases, and evaluate the efficacy of their decision making. The purpose of this manuscript is to introduce the algorithm, lay out the motivation for its inception, and place it in the context of current best-practices in candidate selection. We advocate for a two-step procedure including an initial selection for candidate merit and a subsequent computer-assisted selection from that pool to optimize diversity at the cohort level. The definition of optimal diversity will vary from event to event and the categories of relevant difference are left for each committee to define (see also Section *Limitations*). We suggest that the initial selection for merit could be blind and made by individual committee members in isolation to minimize “groupthink” effects [83]. This step could, for example, incorporate a score given by each committee member to each applicant based on clearly articulated criteria for merit. Because blind selection procedures may lead to unexpected (potentially negative) consequences, these scores should themselves be evaluated for unwanted biases. Recall that disparity diversity cannot always be disentangled from meritorious achievement if demographic factors led to disparate access to opportunity or disparate access to the means to achieve success in the context of equal access to opportunity. The initial assignation of minimum merit thresholds could be set to include individuals who have been presented with fewer than average opportunities and less than average support to mitigate against the objectionable bias introduced by disparity diversity. Any merit selection will likely be subject to a high intrinsic variance, much of which can be accounted for by heuristics and biases in human decision making [41, 50].

As explained above, we therefore suggest translating scores directly into a binary positive or negative decision about admissibility. If the pool of admissible candidates exceeds the number of available spots, the initial candidate selection is then followed by a second step. In the second step the over-sized pool of candidates from the first step is submitted to a computer-assisted selection process from which an appropriately sized, optimally diverse cohort is selected based on the diversity categories the committee has deemed relevant. For instance, some committees may find age to be a relevant diversity characteristic while others may see age diversity as irrelevant. We present a novel algorithm and accompanying software tool called *entrofy* to assist the second stage of the process, framed as an optimization problem over a large number of competing variables. While the main paper lays out the algorithm and discusses its application using both simulations and a case study, the S1 File provide practical advice for using the algorithm and the associated software tool.

## Methods

### Ethics statement

The experimental procedures were approved by the Institutional Review Board at New York University. The questionnaire results referenced in the Discussion section were obtained during (voluntary) online post-workshop surveys in 2016 and 2017, respectively. Workshop participants gave consent online using an IRB-compliant form before beginning the survey.

### Overview

Given a pool of candidates, a collection of attributes describing each candidate, and target proportions of each attribute for the selected set, the goal is to find a cohort candidates whose collective attributes match the target proportions as closely as possible. For this purpose, we define an objective function that measures the relative improvement of adding a given candidate to the pool of previously selected participants, compared to the target selection criteria. Starting either from a group of pre-selected (e.g. invited) participants, or a random position, the algorithm runs sequentially through all candidates and computes the relative improvement of adding that candidate to the cohort set compared to the target categories. The candidate with the highest improvement is added to the cohort set, and the algorithm continues until the pre-defined cohort size is reached.

When two candidates would produce the same improvement, the tie is broken randomly. In practice, for optimization over multiple categories this can lead to overall solutions that are suboptimal, but we show in Results that this problem can be effectively solved by picking the best solution out of several randomized runs of the algorithm.

### Algorithm

Let *S* denote the set of acceptable candidates. Let *a*_*i*_: *S* → {0, 1} denote the indicator function of the *i*^th^ attribute. Given a target set size k∈N, and a set of target proportions *p*_*i*_ for each attribute, our goal is to find a subset *X* ⊆ *S* of size |*X*| = *k* such that

$$\forall i\;:\;\sum_{x\in X}a_{i}\left(x\right)\geq kp_{i},$$ (1)

that is, the selected subset has statistics which meet or exceed the target proportions for each attribute *a*_*i*_. In full generality, there may not be any feasible solution if there are too few candidates possessing the desired attributes. We therefore relax (1) to a maximization of the following objective function:

$$f\left(X\right)≔\sum_{i}w_{i}f_{i}\left(X\right)=\sum_{i}w_{i}\text{min}(kp_{i},\sum_{x\in X}a_{i}\left(x\right)),$$ (2)

where *w*_*i*_ ≥ 0 are non-negative weights, and each *f*_*i*_ measures the number of selected candidates *x* that satisfy attribute *i*, but stops counting after *kp*_*i*_ have been found. The coefficients *w*_*i*_ can be controlled by the user to express the relative importance among competing terms in the objective. It is NP-Hard to find a satisfying solution to (1), as well as to find a solution which maximizes (2), so we do not expect an efficient algorithm to produce exact solutions in all cases [84]. Instead, we will focus on developing an efficient, greedy approximation algorithm with provable guarantees.

The greedy maximization strategy (Algorithm 1) operates by iteratively selecting the point *x* with the maximum *marginal gain* over the current solution *X* ⊆ *S*:

Δf(X,x)≔f(X∪{x})-f(X). (3)

**Algorithm 1** Greedy set-function maximization

1: **procedure** Maximize(*f*, *S*, *k*)

2:  Initialize *X* ← ∅

3:  **while** |*X*| < *k* **do**

4:   $X\leftarrow X\cup \{\underset{x\in S\backslash X}{\text{argmax}}\Delta f\left(X,x\right)\}$

5:  **return** *X*

If the objective function *f* is *monotone* (non-decreasing), non-negative, and *submodular*, then a greedy maximization algorithm is guaranteed to find a solution X* in polynomial time such that *f*(X*) ≥ (1 − *e*^−1^)f*, where f* is the optimal solution value [85]. We demonstrate in the S1 File that (2) satisfies these conditions, and is therefore amenable to efficient, approximate optimization.

### Concave transformation

When optimizing selection over multiple attributes, a single element *x*’s contribution to the objective for each term *f*_*i*_ is either 0 or 1, regardless of how far from the target proportion *kp*_*i*_ the current solution lies. Consequently, when attribute *i* is close to being satisfied while another attribute *j* is far from its target proportion, the greedy selection algorithm cannot distinguish between elements that improve *f*_*j*_ or *f*_*i*_. As a result, the algorithm can produce poor solutions which reach the target proportions for some attributes at the expense of others.

**Fig 1:**
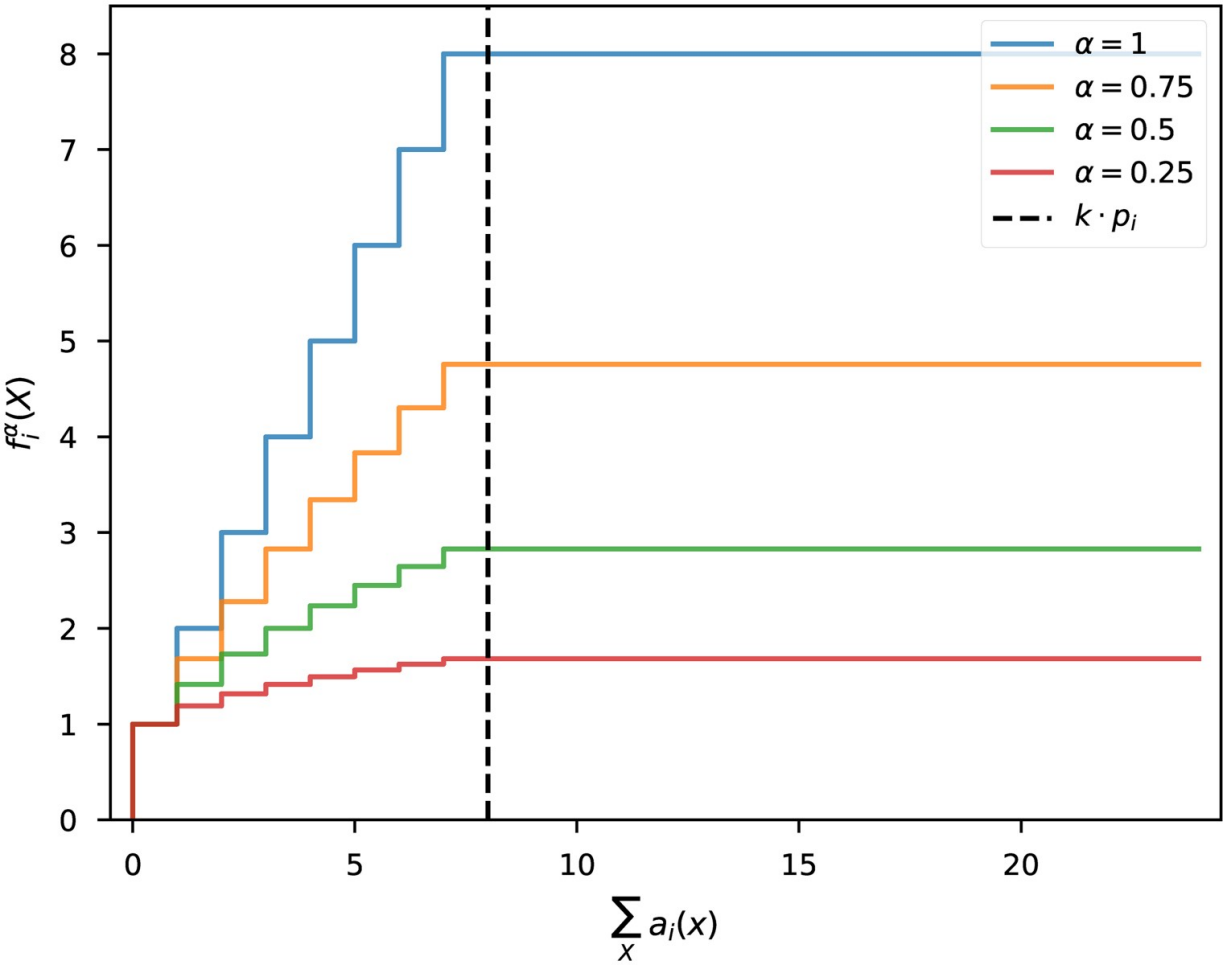
Raising *f*_*i*_ to the 0 < *α* ≤ 1 power leads to diminishing marginal gains as the selected set approaches the target *kp*_*i*_.

This problem can be avoided by applying a concave, monotone transformation to *f*_*i*_, for example, $f_{i}\mapsto f_{i}^{\alpha }$ for some 0 < *α* ≤ 1. Under this transformation, the function remains non-negative, monotone, and concave, and is therefore still amenable to greedy maximization. For values *α* < 1, the marginal gain $\Delta f_{i}^{\alpha }\left(X,x\right)$ diminishes as *f*_*i*_ increases, as illustrated in Fig 1. The greedy selection algorithm is therefore more likely to select elements which improve coverage of attributes that are far from their target proportions.

### Randomization and (near) tie-breaking

The greedy maximization algorithm may have to select among multiple equally good options at each step. Typically, ties are broken arbitrarily by selecting an optimizing candidate at random. When the objective includes multiple competing terms, this can introduce some variance in the objective value of the solutions produced by the algorithm. This variance can be reduced by running the algorithm several times and selecting the result with highest objective value.

Additionally, the algorithm can be made more robust by allowing it to explore slightly sub-optimal local choices. We achieve this by relaxing the greedy maximization to select randomly among elements in the top *q*-quantile of marginal gain. For *q* = 1.0, this reduces to the greedy maximization algorithm.

Algorithm 2 lists the full *entrofy* algorithm, including the concave transformation and randomized near-tie breaking.

**Algorithm 2** The full *entrofy* algorithm

1: **procedure** entrofy(*S*, *k*, {*a*_*i*_}, {*p*_*i*_}, {*w*_*i*_}, *α*, *q*)

2:

$$\text{Let}\;f\left(X\right)≔\sum_{i}w_{i}\text{min}{\left(kp_{i},\sum_{x\in X}a_{i}\left(x\right)\right)}^{\alpha }$$

3:  Initialize *X* ← ∅

4:  **while** |*X*| < *k* **do**

5:   Let *d*(*x*) ≔ Δ*f*(*X*, *x*) for all *x* ∈ *S*\*X*

6:   Let *Q* ≔ {*x* | *x* ∈ top-*q* quantile of *d*(*x*)}

7:   Select *x* uniformly at random from *Q*

8:   *X* ← *X* ∪ {*x*}

9:  **return** *X*, *f*(*X*)

10: **procedure** Entrofy-MC(*n*, *S*, *k*, {*a*_*i*_}, {*p*_*i*_}, {*w*_*i*_}, *α*, *q*)

11:  **for** *i* ∈ 1…*n* **do**

12:   *X*[*i*], *F*[*i*] ← Entrofy(*S*, *k*, {*a*_*i*_}, {*p*_*i*_}, {*w*_*i*_}, *α*, *q*)

13:  **return** the *X*[*i*] with largest *F*[*i*]

### Encoding attributes

The algorithm described in the previous section gives a basic framework with which to select candidates given multiple binary attributes. In this section, we develop extensions of the method to improve its use in practical applications.

#### Non-binary attributes

The algorithm as described above operates only on binary attributes *a*_*i*_, but many quantities of interest take non-binary values. Here, we describe methods to convert non-binary attributes into binary values that can be consumed by the algorithm. We distinguish between two types of non-binary attributes: *categorical* and *ordinal*.

A categorical attribute takes values from a discrete, unordered set of possibilities. Examples of categorical attributes include a candidate’s home institution, area of study, or gender (while we appreciate that gender is not a discrete concept, we advise against modeling gender as a continuous variable here because the proposed algorithmic framework requires an order relation over continuous values). Categorical attributes can be readily converted into binary attributes by applying a *one-hot encoding*, effectively translating a single variable with some *m* possible outcomes to *m* (mutually exclusive) variables each with 2 possible outcomes.

An *ordinal* attribute takes values from a potentially infinite but ordered set, e.g., real numbers. Examples of ordinal attributes include age, publication count, *etc*. Ordinal attributes can be binarized by first quantizing the observed values down to a finite, categorical set, and then applying the previously mentioned one-hot encoding scheme. Quantization thresholds can have a significant impact on the behavior of the algorithm. In the absence of prior knowledge supplied by the user, we partition the space between the observed minimum and maximum values into *m* bins of equal length. When non-binary attributes are binarized, the same weighting coefficient *w*_*i*_ is applied to all corresponding terms in the objective function.

Note that small changes to the boundary positions of histogram bins can produce large changes in the binary encoding and resulting solution. We advise users to be cautious when dealing with ordinal data. In our experience, ordinal values are relatively uncommon, and do not present substantial difficulties in practice.

#### Correlations between attributes

In practice, one might be interested in attributes that are correlated in some way. This could involve intrinsic correlations in the set of candidates, but could also be a design consideration. For example, one might wish to ensure that the selected cohort contains both junior and senior attendees who identify as women. While the algorithm itself cannot take into account correlations between attributes, this is easily solved during engineering of the attributes. For two attributes *i* and *j* with *N*_*i*_ and *N*_*j*_ distinct possible values per attribute, respectively, one can combine both attributes during binarization into a new intersected attribute with *N*_*i*_ *N*_*j*_ possible values. Instead of setting targets on each individual attribute separately, one may then set targets on the possible values for the intersected attribute, which now includes all possible combinations between *i* and *j*.

#### Missing attributes

In some cases, candidates may skip certain survey questions leading to an incomplete response set. This presents no technical challenge for the proposed method, which is inherently robust to missing data. However, it is worth considering the practical consequences of non-response data in the context of cohort selection.

We note that providing responses can only improve and cannot decrease a candidate’s odds of being selected for a given category. For instance, if a particular numerical minority group in a candidate set is systematically skipping a question—for instance, if all very low income people decide not to answer a question about income—they will diminish their individual chances of selection and make it difficult for *entrofy* to produce an optimally diverse cohort. If members of a numerical majority group refuse to respond, they will have less impact on their own chance of selection—they jeopardize their personal chances the more questions they skip—and less impact on overall diversity optimization (unless they opt out as a block). To summarize, skipping questions has a disparately larger impact on those from numerical minority groups. If a sizable proportion of a numerical minority group skips the same question—which could be a small absolute number of people depending on the rareness of the minority status within the applicant pool—this will lessen *entrofy*’s ability to produce a cohort that approximates target distributions set by the selection committee. This is a natural consequence of monotonicity and non-negativity of the objective function (2): attributes *a*_*i*_ contribute non-negatively to the marginal improvement Δ*f* which forms the basis of selection. As a result, it is not possible for a candidate to “game” the system by withholding responses, e.g., if said candidate knows that they belong to a majority subset for a particular attribute.

It is therefore important in any selection procedure to design the survey instrument following best practices [86, 87] with special consideration for known-challenging questions dealing with sex and gender [88], race and ethnicity [89], and any other questions in which responses could place the respondent in a historically stigmatized category. Where specific guidance on questionnaire design by content of question is unavailable, question items might include a write-in option such as “None of these options fits well, I prefer [].” This gives respondents who are willing to respond, but unhappy with the response options, a chance to write themselves in. It gives the committee enough information to make a thoughtful determination about how to incorporate these responses.

## Baseline method

As a baseline for comparison, we implemented a variant of the classic “bounded-greedy” diversity maximization method by Smyth and McClave [76]. This method was developed for diversification of search and recommendation, where the selected set must be simultaneously relevant to a *query*, but also have maximal variance. In our setting, we assume all candidates in *X* are of equal “relevance” (i.e., have all passed an initial merit-based review), so the method simplifies to maximizing diversity of the selected set.

Smyth and McClave define diversity as the average of all pairwise dissimilarities within the selected set, which is equivalent to sample variance when candidates are compared by Euclidean distance. For our application, each candidate is represented by a binary attribute vector, and we compare candidates *x*, *y* by their Jaccard similarity:

$$J\left(x,y\right)≔\frac{\sum_{i}\text{min}\left(a_{i}\left(x\right),a_{i}\left(y\right)\right)}{\sum_{j}\text{max}\left(a_{j}\left(x\right),a_{j}\left(y\right)\right)},$$ (4)

that is, the ratio of the number of common attributes between candidates to the total number of attributes exhibited by either candidate. Diversity is then quantified as the average dissimilarity between selected candidates

$$f\left(X\right)≔\underset{x,y\in S}{\text{mean}}\left(1-J\left(x,y\right)\right).$$ (5)

Selection of the set *X* then proceeds much as in *entrofy*: candidates *x* are selected greedily to maximize the marginal improvement in *f*(*X* ∪ {*x*}). As with *entrofy*, we extend this method to support randomized tie-breaking and top-quantile selection to ensure a fair comparison between methods.

We note that the Smyth-McClave method is intended to maximize *total* variance, but has no explicit incentives to approximate target proportions for each individual attribute, so we do not expect it to perform well in our particular application.

## Results

### Experiment 1: Simulating a single solution with varying noise

We performed several controlled experiments with simulated data sets to demonstrate that our algorithm can successfully recover a solution, i.e., an optimal set of participants embedded in a larger set of random data. We simulated a hypothetical data set of candidates with two categories, each of which comprises two attributes (denoted “yes” and “no”). For each simulated data set, we first generate an optimal solution (which we call the *cohort)* to be planted in the data set based on a set of target proportions, *f*_target_, for each category, to ensure that an optimal solution could in principle be found in every simulation. The objective of these simulations was not to present realistic circumstances (where target proportions can be far from the properties of the candidate set and an optimal solution might not exist), but to explore situations where the algorithm fails when in principle, it should be able to find a solution. Note, therefore, that in what follows, the term *target proportions* applies both to the attribute distribution of the simulated cohort which is to be recovered, as well as the targets applied to *entrofy* in order to recover this cohort. To each simulated cohort, we then added a number of candidates with attributes that are randomly selected based on some (other) probability distribution over the attributes, *f*_random_. These additional candidates (which we denote *distractors*) effectively act as a type of noise. Together, the simulated cohort and the distractors from the *candidate set*, i.e. the set of candidates who applied and from which the cohort is to be selected. In realistic situations, ensembles of candidates might be imbalanced with respect to the target proportions; indeed, it might be a stated goal of the selection procedure to address imbalances. Hence we perform simulations with data added to the cohort with properties both similar and very dissimilar to that cohort. In order to understand whether the performance of *entrofy* changes as a function of the size of the cohort and the candidate set, we vary both the size of the cohort to be found, as well as the number of distractors added to each cohort.

### Set-up

We varied target proportions *f*_target_ between 0.1 and 0.5 (because our categories are binary, they are symmetric about 0.5). We also varied the probability distribution of the attributes of the distractors, *f*_random_ between 0.0 and 0.5, since it is in principle possible that members with certain attributes exist only in the solution and not elsewhere in the candidate set. We varied the number of participants cohort, *n*_out_ between 10 and 200, since we deem it unlikely that the algorithm will be used for significantly larger target sets. We let the number of distractors added to this cohort, *n*_random_, vary between 1 and 1000. We also varied the parameter *α* used in the objective function between 0.1 and 1.0. For each combination of parameters, we performed 100 simulations, leading to a total of 24.3 × 10^6^ simulations. For each simulation, we ran *entrofy* exactly once. We then computed the value of the objective function for the cohort embedded in the data, and calculated the difference between the cohort found by *entrofy* for this candidate set and the objective on the solution we embedded. When this difference exceeds 0, we deemed the solution found by *entrofy* to be a failure. When the difference is 0, this indicates that *entrofy* found either the planted cohort, or one of equivalent objective value, which we took as success.

### Results

**Fig 2:**
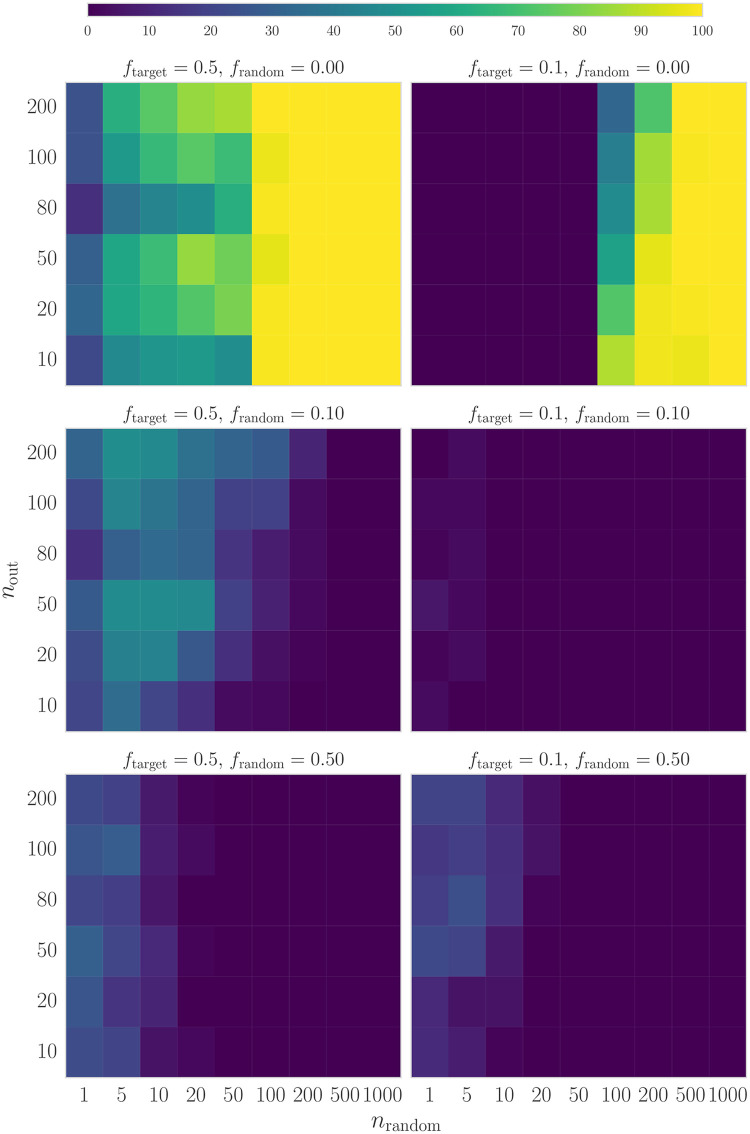
Failure rates for simulations with different parameters. Colours indicate the number of failures out of 100 total simulations per bin. On the horizontal axis, we plot the number of distractors added to each cohort *n*_random_ to simulate noise in the data. On the vertical axis, we show the number of participants in the cohort to be found *n*_out_ (equivalent to the size of the cohort embedded in the candidate set). The size of the candidate set for each simulation is the sum of the size of the cohort, *n*_out_, and the number of distractors, *n*_random_. For all simulations, for one attribute the target proportions of the embedded cohort as well as the distribution of the distractors of one category are kept constant at 0.5, while values of both target and distractor proportions are varied for the other category. The left-hand column shows results where the target proportion for the second category is kept constant at 0.5, while the proportion of distractors is varied between 0 and 0.5. In the right-hand column, we show similar plots for a target proportion of 0.1 instead.

In Fig 2, we present the results of our simulations. Here, we kept the target proportions as well as the proportions of the distractors of one category constant at 0.5 and varied the proportions for the planted cohort and the distractors of the other attribute to explore the effect both have on the solution. In all cases, we used *α* = 0.5. We find that the strongest failure mode occurs when the proportion of distractors for this second attribute is *f*_random_ = 0.0. In this case, the embedded cohort is the only set of participants that will satisfy the targets provided to *entrofy* and yield an acceptable value of the objective function. Because we use quantiles for tie-breaking when candidates have very similar attributes, this makes finding the optimal solution near-impossible when the set of candidates lacking the attribute in question is very large. Here, the algorithm will almost always fail on a single try, even if the target proportion of that attribute is as low as 0.1. There appears a fairly sharp transition when the candidate set includes 100 or more distractors, no matter the size of the cohort to be found. For ensembles larger than that, the algorithm will fail nearly 100% of the time. However, we caution the reader that the exact position of this transition is not well determined, since the grid used in exploring parameter is fairly coarse (and uses steps of either 0.1 or 10 for almost all parameters).

When the distractors do not entirely lack representation in one attribute, i.e. *f*_random_ > 0, the rate of failure is lowest when distribution of the distractors matches the distribution of the cohort, and highest when the distribution of distractors and embedded cohort are most dissimilar. In all cases, failure rates are <50% on a single run of *entrofy* on a given candidate set, and failure rates are higher when the number of distractors added to the embedded cohort is small compared to the size of the cohort. This, too, is related to the number of equally optimal solutions in the data set: when the number of distractors added to the cohort is large, and distributions over cohort and distractor are not too dissimilar, there will be a number of possible solutions, and the probability of the algorithm finding one of them is comparatively large. Conversely, when there are only few distractors added to the cohort embedded in the data, that cohort is likely the only acceptable solution for *entrofy* to find, and it will be less probable that the algorithm finds exactly that solution. As expected, this effect is exacerbated if the properties of the distractors and the cohort differ strongly.

The behaviour described above is largely independent of the parameter *α*. For *α* between 0.1 and 0.5, the results from the simulations are largely consistent. Only when *α* = 1, the failure rate increases significantly, overall by about 5 failures for each combination of parameters. This is expected, since *α* changes the shape of the objective function in a way that will make it slightly harder for the algorithm to find the optimal solution when *α* = 1. Thus, we recommend *α* = 0.5 as a reasonable value reliably returning optimal solutions.

**Fig 3:**
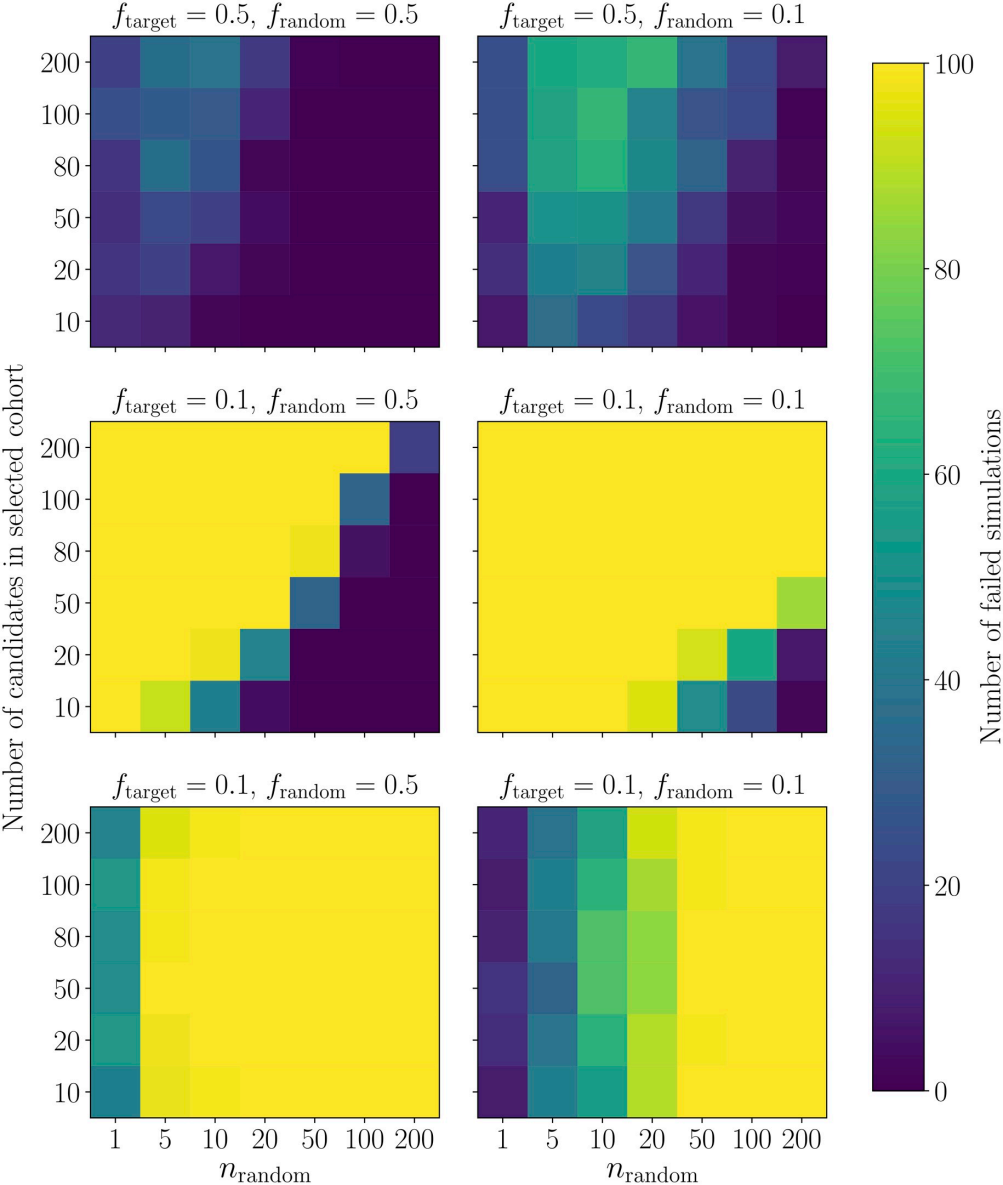
As for Fig 2, we present failure rates as a function of the cohort size (vertical axis) versus the number of distractors (horizontal axis), for the Smyth and McClave baseline algorithm from [76]. We explore the behaviour of the baseline algorithm both in terms of whether it can recover maximally diverse cohorts (upper four panels), and whether it can recover imbalanced cohorts. We look at four particular cases: in the upper left panel, a case where both the embedded cohort and the distractors are balanced with respect to the two attributes in the shown category, i.e. the entire candidate set has a uniform distribution for the relevant attributes. In the upper right, a case where the embedded cohort has uniform targets across both attributes, but the distractors added are skewed with *f*_random_ = 0.1. In the middle left, the converse case, where the embedded cohort is skewed, but the distractors balanced, and finally in the middle right a case where both the embedded cohort to be selected and the distractors have a highly skewed distribution. We note that the middle row is a special case: here, *f*_target_ only corresponds to the proportions of the embedded cohort, while “success” for these two panels is defined as recovering maximally diverse cohorts, as this particular algorithm is designed to do. The bottom row shows the same simulations as the middle row, but presents successes and failures when the targets that generated the embedded cohort are applied instead of the balanced targets that are part of the baseline’s objective function. The baseline method can recover embedded cohorts if (1) the targets for those cohorts are maximally diverse (upper row), and (2) if the targets are skewed, but there is a number of maximally diverse distractors that is much larger than the cohort to be selected. The baseline method can generally not recover cohorts with imbalanced targets, unless the overall candidate set is so imbalanced that there is no choice but to return a cohort with skewed attribute distribution (left-most columns of the two panels in the bottom row).

We compared *entrofy*’s ability to return cohorts that approximate a given set of target distributions with our baseline algorithm [76]. In Fig 3, we present analogous simulations to Fig 2 (100 simulations per bin, for different *f*_target_ and *f*_random_), but using the Smyth and McClave algorithm for generating cohorts. Because the objective function for the baseline method is defined in terms of maximally diverse cohorts, not in terms of the target distributions we would like to recover, we apply a somewhat different metric. In order to quantify similarity between a given target distribution and the cohort recovered by the baseline method, we define a distance between a candidate set and the targets as

$$d\left(X\right)=\frac{1}{N}\sum_{i=1}^{N}\frac{1}{n_{i}}\sum_{j=1}^{n_{i}}|\sum_{x\in X}\frac{a_{i,j}\left(x\right)}{\left|X\right|}-p_{i,j}|$$ (6)

for *N* categories with *n*_*i*_ possible attributes each. If *X* denotes the selected cohort, then this quantity measures the average distance between the targets and the selected cohort and is ideally *d*(*X*) = 0. The same quantity can be computed for *X* = *S*, i.e., the deviation of the entire set of candidates from the targets before selection. This allows us to explicitly compare performance in terms of recovering an embedded cohort as a function of the attribute imbalances within that cohort.

We note that the objective function agrees with the targets chosen by the selection committee only if these targets indicate maximum diversity, i.e. *f*_target_ = 0.5. For simulated cohorts where *f*_target_ is far from parity, we test two separate cases: (1) the case that the Smyth and McClave algorithm is designed to do, i.e. find a cohort of maximum diversity (which will be dissimilar from the cohort we embedded during simulation). We then also test the baseline algorithm’s ability to recover the originally embedded cohort.

We find that the baseline algorithm is generally competent at returning maximally diverse cohorts when the target distribution for these cohorts is uniform (Fig 3, upper row of panels), or if the candidate set overall exhibits fairly uniform target proportions (Fig 3, middle left panel), even though the embedded cohort may have imbalanced proportions across an attribute. This is the case, for example, when the embedded cohort is small compared to a large set of distractors with a balanced distribution across attributes. In the middle left panel of Fig 3, there is a sharp diagonal transition between cases where the baseline algorithm always fails, (upper left of the panel), and where it always succeeds (lower right of the panel). These correspond to cases where there is a large cohort with imbalanced proportions embedded in a small set of distractors with balanced attributes, which will always fail to return maximally diverse cohorts, and cases where a small, imbalanced cohort is embedded in a much larger set of distractors, which will allow the baseline method to find and return maximally diverse cohorts. In practice, however, this is at odds with our goals: in the lower four panels, we embedded imbalanced cohorts specifically to see whether the algorithm could recover these cohorts. In the bottom row of the plot, we therefore test whether the baseline algorithm can recover cohorts with the expected target proportions of a heavily imbalanced embedded cohort. We find that the baseline will only return appropriately imbalanced cohorts when it is forced to do so (bottom row of panels in Fig 3), for large imbalanced cohorts embedded in a small set of distractors (left-most columns of those panels). Here, there simply are not enough candidates for the algorithm to return maximally diverse cohorts, and it thus returns cohorts close or equal to those expected by the equally imbalanced targets: for imbalanced targets, the baseline method is therefore only ever successful by accident. This is unsurprising, given that the objective function maximizes diversity within the cohort, but it implies that the use case of the baseline is strongly restricted compared to that of Entrofy, which can recover cohorts for a range of balanced and imbalanced target distributions.

**Fig 4:**
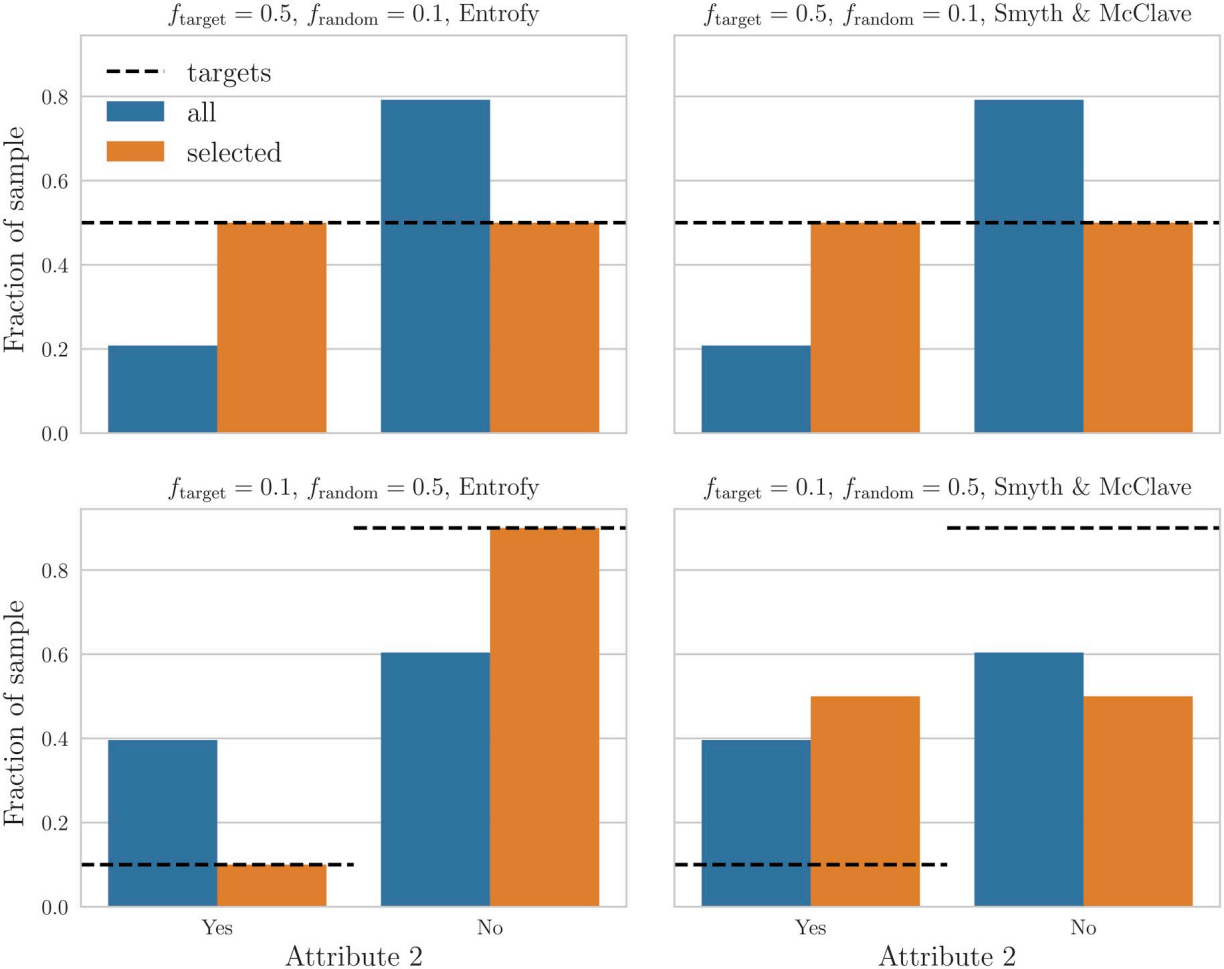
An illustration of the different behaviour of the *entrofy* algorithm compared to the baseline. Here, we show the results of a single simulation per panel, closer to what one might see in reality. For all four panels, combined a cohort of *n*_out_ = 50 participants with *n*_random_ = 200 distractors. We show the distribution of the candidate set across possible attributes in blue, and the distribution of the selected cohort in orange. To guide the eye, black dashed lines show the target distribution to be approximated by the selected cohort. Both panels in the left column present results returned by *entrofy*, while the right column presents corresponding results from the baseline algorithm. In the top two panels, we show a simulation that included a cohort with a balanced distribution of *f*_target_ = 0.1 for one attribute added in, and a skewed set of distractors, *f*_random_ = 0.1. The bottom two panels show the reverse case, with an imbalanced cohort, *f*_target_ = 0.1 and a balanced set of distractors, *f*_random_ = 0.5. Both *entrofy* and the baseline algorithm can approximate uniform distributions as shown in the upper panels. However, while *entrofy* can approximate non-uniform targets (lower left), our baseline algorithm will always attempt to return maximally diverse cohorts, and thus not be able to approximate our skewed targets (lower right).

This is underscored by the results in Fig 4, which presents a different view on two individual instances of the simulations in Fig 3. Here, we show results for *entrofy* (left) and the baseline (right) for two different types of simulations: one where the target distributions are uniform, but the distractors follow a skewed distribution (top), and another where the target distributions are skewed, but the added in distractors are uniform. In both cases, we generate a cohort of *n*_out_ = 50 participants, and a set of *n*_random_ = 200 distractors to approximate a realistic workshop participant selection scenario. Both *entrofy* and the baseline algorithm can recover a balanced cohort, even when the overall distribution of candidates is very skewed with respect to the target to be approximated. Conversely, while *entrofy* can recover cohorts with a skewed distribution of attributes (Fig 4, lower left panel), the baseline algorithm is designed to return maximally diverse cohorts, and is therefore unable to return a cohort with the desired characteristics.

### Experiment 2: Simulating multiple runs

In practice, running *entrofy* several times on the same data set and choosing the solution with the maximum value of the objective function is a simple way to mitigate failures on single runs. While this increases runtime, the additional computational cost is small enough on all reasonable data sets (up to at least 1500 candidates in the candidate set) to merit the increase in accuracy.

#### Set-up

In order to test how many simulations are generally necessary to make success highly probable, we picked a case where nearly half of the simulations failed and tested for success as a function of the number of times *entrofy* is run on a single data set, *n*_trials_. We chose a case with a cohort of 100 participants and target proportions of 0.5 for each attribute in both categories. Again, we simulated a solution with these parameters, and then added 5 additional candidates to the set distributed with one attribute split 0.1 (“yes”) and 0.9 (“no”), the other 0.5 for “yes” and “no” both. In our original simulations with *α* = 0.5, this led to 49 failures, the highest rate of failure in simulations with this particular combination of target and distractor distributions. As described above, we compute the objective function for each embedded solution, and subsequently compare with the score returned by *entrofy*. In this experiment, however, we vary the number of trials used in *entrofy* for computing the objective score, between 1 and 200, and run 100 simulations for each value of *n*_trials_.

#### Results

**Fig 5:**
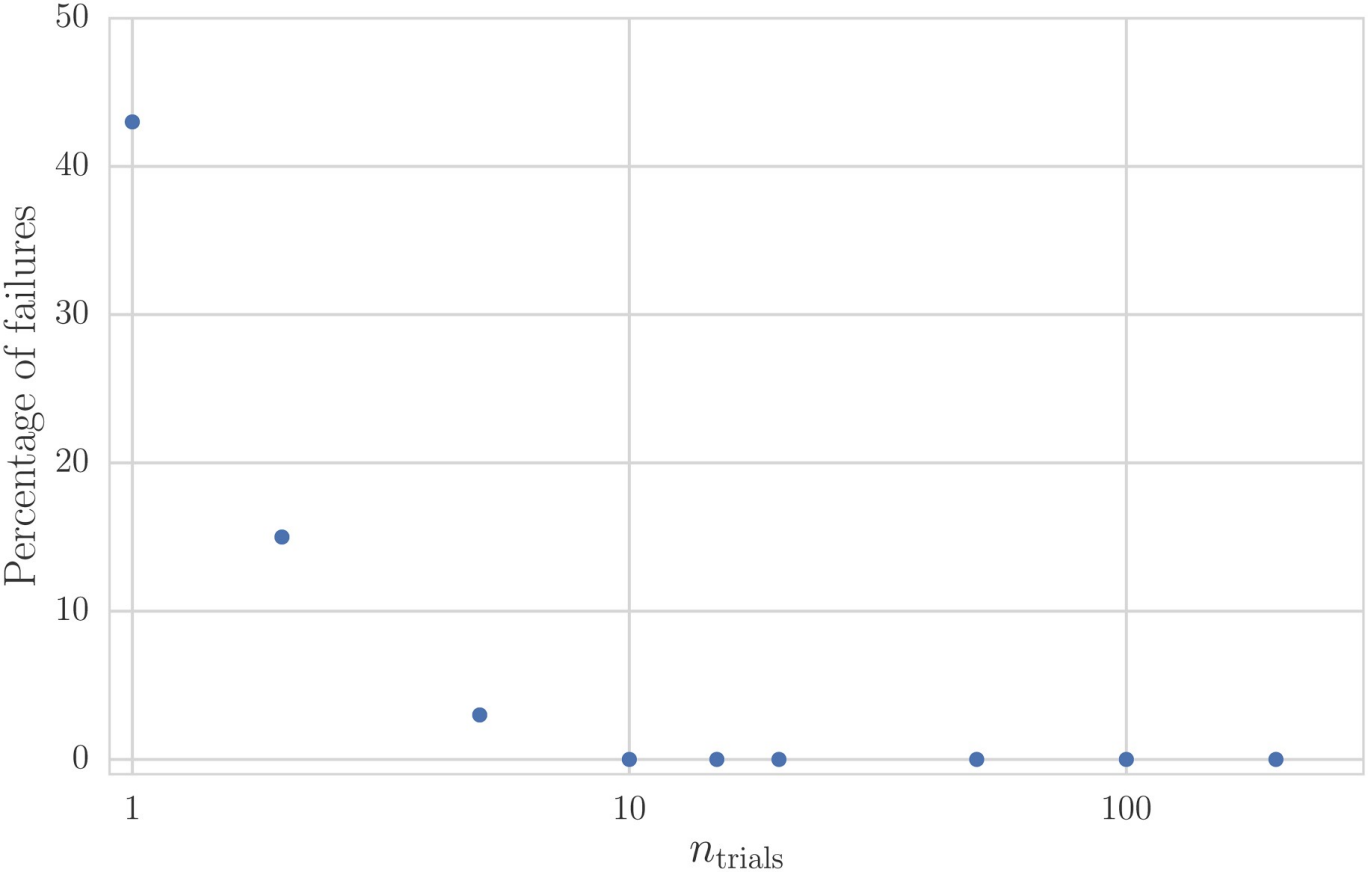
The percentage of failed *entrofy* runs versus the number of trials used to find the optimal solution. This simulation uses an optimal solution of 100 participants in a set of 105 candidates, where the target proportions of 0.5 for the defining attribute are dissimilar from those of the set of distractors added to the cohort (*f*_random_ = 0.1). We choose *α* = 0.5 and compute the percentage of failures as a function of the number of *entrofy* runs *n*_trials_ on each simulated data set, using 100 simulations for each value of *n*_trials_. For 10 or more trial runs, *entrofy* almost always finds the optimal solution.

In Fig 5, we present the results of these simulations. Consistent with our previous results, nearly half of the *entrofy* runs fail to find the optimal solution when the algorithm is run only once. As soon as multiple trials are used, however, the failure rate drops sharply and reaches 0 at around 10 iterations. Thus, in practice, allowing *entrofy* to run Â 10 times and report the best out of those runs allowed us to successfully find the embedded optimal solution in all test cases. In general, this value might depend on the number of distractors, the number of categories and the overall properties of the candidate set compared to the targets, thus in practice very complex selection procedures might benefit from running additional trials.

### Case study: Astro hack week

Astro Hack Week is a five-day workshop started in 2014 that recurs annually, with six completed hack weeks so far. It focuses on data-intensive research in astronomy and has multiple goals: education of the community in the most recent data analysis methods, communicating and encouraging best practices for research, fostering active collaborations and peer learning within astronomy and between astronomy and adjacent disciplines, and networking (for more details, see [90]). Because Astro Hack Week includes a significant project-work component, where impromptu teams get together to work on a particular scientific or technical problem, the effects of different types of diversity on outcomes, in particular on participant satisfaction as well as group cohesion and learning, are of critical importance to the success of the workshop, and need to be understood in order to design effective interventions that enable group work. Much of the project work, by its nature as a *hackathon*, requires innovation and creativity, suggesting that diversification across types of knowledge and career stages should lead to positive outcomes if the diversity-cognitive resources theory holds. On the other hand, diversification across surface-level characteristics such as gender or cultural background may lead to negative effects due to participants implicitly or explicitly categorizing group members based on interpersonal similarities, generating ingroup/outgroup effects that may disrupt team processes and engender intergroup biases that may be pronounced for minority participants. Because Astro Hack Week is routinely oversubscribed by a factor of >2, it is generally possible to manage diversification across different categories intentionally, which makes cohort selection a central problem during conference organization. We aim to admit diverse cohorts across deep-level characteristics such as programming experience and knowledge in data science-relevant areas in order to maximize positive effects related to the diversification of cognitive resources. With respect to surface-level characteristics such as demographic categories, our aim is to admit diverse cohorts in accordance with our objective of increasing the participation of researchers from underrepresented backgrounds in data-intensive astronomy. The mitigation of potentially adverse effects on participant satisfaction and group cohesion due to surface-level diversity is the core objective of the comprehensive workshop facilitation plan enacted throughout the workshop. This plan, subject to annual design review and evaluation, aims to enable all attendees to engage fully with the workshop, but pays particular attention to participants who are most likely to experience adverse effects due to outgroup membership.

For the events in 2016 and 2017, we adopted the approach advocated in this paper: a staggered procedure of meritorious selection followed by optimization based on the goals of the conference. Applicants were informed about the purpose and use of the data being gathered, then filled out a survey including questions about their motivation and goals for Astro Hack Week, their data science-related skills and (optionally) their demographic background.

In the first candidate selection step, we performed a blind merit assessment based on candidates’ responses to questions about their motivation and goals for Astro Hack Week. Candidates names and demographic information were unavailable to reviewers during the first step. We note that Astro Hack Week is idiosyncratic in that the workshop‘s’ extraordinarily broad scope translates into a lack of formal merit criteria to be enacted during the first step of the procedure. Because Astro Hack Week’s core component is free-form project work with an emphasis on creativity and innovation, and the category of admissible projects is very wide, we have elected to treat merit selection as a stripped-down process that primarily removed spam responses submitted to our registration form, and candidates whose goals were entirely unrelated to that of the workshop. In total, we selected all 110 candidates in 2016 and 155 out of 160 candidates in 2017.

The nature of the workshop requires approximating a carefully designed set of target proportions for a number of different categories, including demographic diversity (race/ethnicity and gender in both years, as well as geographic location in 2017), knowledge of relevant data analysis methods (machine learning, statistics and programming), academic seniority (including all academic ranks from undergraduate students to senior faculty, as well as non-academic roles), and previous attendance at this or a similar event. In total, we optimized over 8 categories (2017: 9 categories), with between 2 and 6 possible options each. We pre-selected 11 candidates (2017: 9 candidates) including the scientific organizing committee and selected the remaining cohort of 38 participants (2017: 45 participants) using the algorithm defined in Methods. In order to quantify similarity between our targets and the distribution of candidates in both our candidate set as well as our selected cohort, we use the distance metric defined in Eq 6.

**Fig 6:**
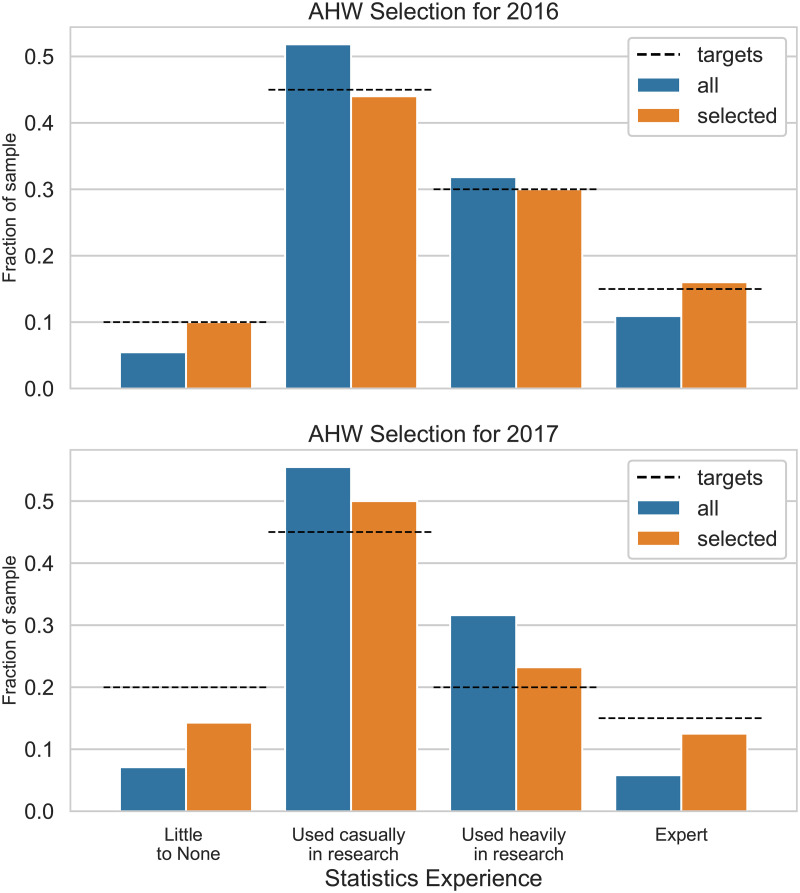
This figure shows responses to a question from the registration form asking candidates to self-report their statistics knowledge before the workshop, for both the 2016 workshop (upper panel) and the 2017 workshop (lower panel). In blue, we plot the fraction of candidates for each answer in the candidate set before performing automated selection. For comparison, the fraction of candidates from the selected cohort for each answer is shown in orange, and the targets set by the organizing committee based on the workshop goals is plotted as a dashed black line. In both cases, the selected cohort is notably closer to the pre-defined targets than the original candidate set, though the effect is much stronger in the 2016 case. The distance between the set of selected candidates and targets is *d*(*X*) = 0.004, compared with *d*(*S*) = 0.043 for the full set of candidates for the 2016 workshop, whereas the distance between selected candidates and targets is *d*(*X*) = 0.04 for the 2017 workshop, with an original distance between targets and candidate set of *d*(*S*) = 0.11.

In Fig 6, we present the results of the selection procedure using one particular category as an example. We find that for both workshops, the set of acceptable candidates (or a random selection thereof) diverges significantly from the ideal cohort as defined by our workshop goals. For this particular category, we find an initial distance between the set of candidates and the targets of *d*(*S*) = 0.043 in 2016, and *d*(*S*) = 0.11 in 2017. In the selected cohort, the distance is reduced by more than a factor of 10 to *d*(*X*) = 0.0041 (2016), but only by about a factor of 3 to *d*(*X*) = 0.04 in 2017. The latter is due to the fact that despite the larger selected cohort in 2017, the overall divergence between the candidate pool after step 1 and targets was much larger, to a point where it became impossible to find a solution close to the targets.

**Fig 7:**
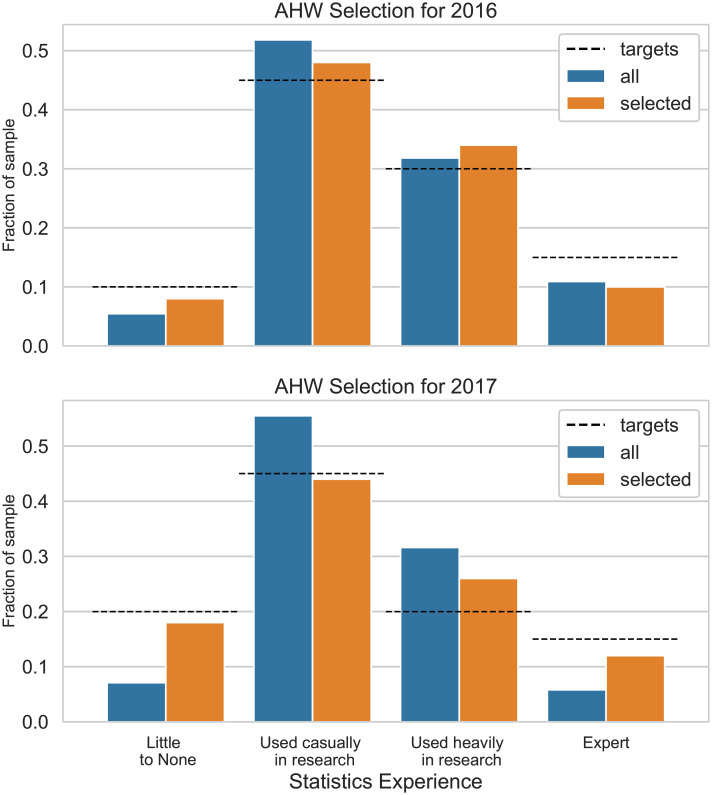
Equivalent figure to Fig 6, but with results from the Smyth and McClave baseline algorithm [76] in orange instead of *entrofy*. Because our targets are not maximally diverse–we aim to admit significantly fewer candidates with no knowledge in statistics than with some or expect knowledge in statistics–the diversification using the Smyth & McClave bounded greedy algorithm is farther away from the targets in 2016, since it aims to select cohorts that are maximally diverse across all categories. For 2017, the outcomes are somewhat more equivocal, partly because targets were slightly more similar across different answers, and because the candidate set was more constrained in a number of different categories.

For comparison, we also selected a cohort using the baseline algorithm [76], and find a distance between that cohort and the targets *d*(*X*) = 0.026 in 2016 and *d*(*X*) = 0.032 in 2017. While in 2016, the baseline method fares worse than *entrofy* in selecting a cohort that adheres to the targets we set, in 2017 the baseline method provides slightly better scores than *entrofy* (see also Fig 7 for a comparison of candidate set to a cohort selected by the baseline method). There are a number of potential reasons for this outcome: as we have mentioned above, the overall divergence between candidate set and targets was much larger in 2017, and those targets may have pushed *entrofy* to select candidates that help fulfil targets in other categories, at the penalty of making the outcome for this particular category slightly worse. In this context, it makes sense that an algorithm that generally pushes the selected cohort towards a similar level of diversity across all categories to do slightly better.

In order to explore this question further, we repeat this analysis using all categories, which yields distances for the candidate set to the targets of *d*(*S*) = 0.074 in 2016 and *d*(*S*) = 0.148 in 2017. Note, however, that the 2017 data set includes an additional category, geographic location, for which we had set ambitious targets not supported by the set of candidates. Even without inclusion of that category, the average distance between candidate set and targets is still *d*(*S*) = 0.12, almost twice as high as for the 2016 set, indicating that perhaps the 2017 workshop attracted a slightly different population from the earlier workshop. Part of this might be explained by the much higher fraction of graduate students who applied in 2017 (62% compared to 50% in 2016) and the lower target for that particular category set (0.3 in 2017 versus 0.4 in 2016).

Overall, *entrofy* found a selected cohort in 2016 that reduced the distance between the candidate pool after step 1 and targets to *d*(*X*) = 0.028 in the selected cohort. While there seems to be no optimal set of participants in the data, the application of *entrofy* has resulted in selected cohort that is a considerably closer fit to the desired cohort targets than we would have gotten if we selected a cohort that matched the properties of the applicant pool. In contrast, the baseline method only achieves a distance of *d*(*X*) = 0.055, almost twice as high as that found with *entrofy*. We trace this back to the distribution of targets, which in many cases correspond to a very non-uniform categorical distribution across possible responses. This is naturally hard for an algorithm designed to drive outcomes towards maximally diverse cohorts. In 2017, conversely, the overall change in distance is much smaller, by less than a factor of 2 to *d*(*X*) = 0.088. Much like for the single category above, for the 2017 dataset the baseline method achieves a comparable distance overall as *entrofy*, *d*(*X*) = 0.089. The differences between 2016 and 2017 can be explained by the constrained nature of the the candidate pool after step 1: with only an over-subscription of a factor of ∼2 and for some categories a large divergence between the candidate pool after step 1 and targets, an optimal cohort may simply not exist. We note that overall our targets for 2017 were somewhat more uniformly distributed across attributes compared to 2016, a case where we expect the baseline algorithm to perform comparably to *entrofy*.

The concave transformation weights participants higher if they improve a category still far from the target, sometimes at the expense of introducing small deviations in other categories. Note also, if selected candidates decline to attend, re-selection can also be performed with *entrofy*). If candidates preferentially drop out in certain categories that are underrepresented, this may exacerbate the distance between the selected cohort and pre-defined targets.

## Discussion

We have presented a cohort selection procedure that aims to support committees in their decision making processes through an algorithm that allows for optimizing towards diversity targets across a set of relevant categories. The key purpose of this procedure is to somewhat decouple the cognitive tasks of assessing merit and cohort composition and enable transparency for audit and evaluation.

In this section, we briefly comment on anecdotal outcomes from Astro Hack Week, discuss limitations of the approach and highlight avenues for future work.

### Algorithmic cohort selection in context: Observations from astro hack week

There is now a wealth of available research, especially from the employment literature, that recommends making assessment and decision processes as structured and quantitative as possible to facilitate better decision outcomes [91]. This allows committees to be accountable to themselves and to the candidates they are assessing. Because goals and requirements may differ for different scenarios, experimentation with strategies, questions, weights and targets should be encouraged, as should be critical evaluation both during and after the selection. This is especially true for recurring selection events (e.g., annual conferences), where cohorts can be compared laterally. *entrofy* fits within this framework in three ways, providing benefits to selection committees beyond the output of the algorithm.

First, it helps committees develop the data set and the language around decision processes to enable evaluation and discussion. Because *entrofy* requires committees to explicitly attach numerical targets and weights to attributes, discussions shift toward the goals and requirements of the conference, workshop, or degree program. In particular, committees discuss how to translate their aims into questions asked of candidates and to establishing targets or weights for *entrofy*. This is not unique to *entrofy*, but part of a larger cultural shift toward making evaluation procedures more quantitative, based on a wealth of research suggesting that quantitative procedures tend to be less biased [92, 93]. Drawing on Astro Hack Week as an example, participant questions about computer programming skill have shifted from being broad and undirected (e.g., *how many years of programming experience?*) to more fine-grained, objective, and well-calibrated questions that people can realistically self-report (e.g., whether they have written certain programming constructs like functions or classes). Similarly, the procedure encouraged the committee to critically think about the results of the selection, and allowed it to first visualize cases of interest (e.g., whether there were correlations between career stage and gender) and subsequently discuss the inclusion of an intersectional category in the selection to ensure representation of gender minority participants at all career stages. Detailed evaluations and discussions about workshop goals, in turn, have helped clarify the selection process, but unexpectedly aided the committee in other crucial tasks like program decisions. Overall, it has provided a framework for critical post-hoc evaluation, comparing selection procedures and workshop outcomes over successive years to improve the process or experiment with alternative strategies.

A second positive side-effect is that the associated data collection and visualization can help the committee identify potential biases inherent in applicant pool and selection procedure. With respect to Astro Hack Week, the committee used data visualizations made as part of the *entrofy* process to identify a lack of candidates from underrepresented racial and ethnic minorities. This in turn led to targeted outreach efforts toward those groups, as well as directed fund-raising efforts to aid students from disadvantaged backgrounds and smaller institutions.

Third, *entrofy* provides algorithmic transparency and accountability for a significant part of the selection procedure. In the case study of Astro Hack Week during which participants were told that *entrofy* had been part of the selection procedure, an online survey was administered at the end of the workshop to gauge participants’ attitudes. 82% agreed with the statement “I think that using an algorithm for selection makes the process more transparent” (see Table 1). Similarly, 81% reported agreement with the statement, “I believe the selection for Astro Hack Week was fair”. Because academic meetings are an important venue for discussion, collaboration, and career development, both conference organizers and research communities have a vested interest in making conferences and workshops equitable to candidates from all backgrounds and at all stages in their careers. An overwhelming 97% of respondents in our case study agreed with the statement, “I think Astro Hack Week benefitted from a wide range of backgrounds and experiences of the attendees.” Participants appear to view diversity of the group from a cognitive-resource perspective, but may not be sensitive to adverse effects, especially if these effects are only experienced by a subset of participants. [27] suggest that *diversity mindsets*, i.e. the beliefs and attitudes team members hold with respect to diversity in the context of team work, play an important role in determining the effects of diversity on team outcome. The similarity of mindsets among members (sharedness) and the awareness of that similarity are expected to mediate that relationship. It may be interesting to explore the views of our participants on team diversity, the sharedness of these views and the effect on workshop outcomes in more detail in future surveys.

**Table 1:** 

	Negative outcomes of using *entrofy*?		Positive outcomes of using *entrofy*?		
	*I think the wide range of backgrounds made the meeting too unfocused*.	*I was more uncomfortable because attendees came from a range of backgrounds*.	*I think Astro Hack Week benefitted from a wide range of backgrounds and experiences of attendees*.	*I think that using an algorithm for selection makes the process more transparent*.	*I believe the selection procedure for Astro Hack Week was fair*.
Strongly Disagree	14 (42%)	12 (36%)	0 (0%)	0 (0%)	1 (3%)
Disagree	9 (27%)	6 (19%)	0 (0%)	0 (0%)	0 (0%)
Somewhat Disagree	5 (15%)	3 (10%)	1 (3%)	3 (10%)	0 (0%)
Somewhat Agree	4 (12%)	5 (15%)	4 (12%)	4 (13%)	2 (6%)
Agree	0 (0%)	4 (12%)	10 (30%)	9 (27%)	11 (33%)
Strongly Agree	1 (3%)	3 (9%)	18 (54%)	14 (42%)	14 (42%)
Don’t Know	NA	NA	NA	3 (10%)	5 (15%)

Respondents to a post-event evaluation survey indicate their level of agreement with the statements in the header. *N* = 33; response rate 66%

The underlying justification for using a two-stage selection process is likely to apply to other situations where decision makers must select a reasonably large subset of candidates, including cohort hiring, speaker selection for seminar series, and degree program admissions procedures. Splitting the selection into two steps and framing the second part as a clearly defined mathematical procedure provides this transparency: if a candidate is rejected, that decision can be traced back to either the merit-based selection stage or the randomized tie-breaking stage (*entrofy*).

### Limitations

The procedure laid out in this paper aims to improve the transparency and auditability of selection procedures by requiring selection committees to make their goals and selection criteria explicit and quantitative. In the second step, an algorithm is used to support human decision making in a task that tends to require larger cognitive resources than can be supplied by any human (or small group of committee members). This approach has a number of limitations, however, both at the algorithmic and practical level.

At the most fundamental level, it is worth noting that no algorithm is free of bias: as human-generated constructs, algorithms and procedures, including the one presented here, encode and reflect the beliefs and biases of their creators [42]. The second, automated step of the process makes an assumption that the diversity attributes of interest are inherently categorizeable. This may not be true for diversity criteria in Harrison and Klein’s *separation* type [31], encoding for example differences in beliefs, values or attitudes, which may fall onto continua rather than categories.

Careful human judgment is required at several points in the procedure, most notably in the initial selection for merit, the selection of attribute categories to be included in the algorithm, and establishing the size of target proportions. Even for blinded reviews in the first stage, assessors must be vigilant in checking their biases or the set of acceptable candidates may be distorted in undesirable ways. The merit review is a crucial step in the procedure and its design will strongly affect outcomes of the cohort selection. The procedure presented here makes intrinsic assumptions about the separability of merit criteria from categories of diversity, which may be untrue in practice. Especially diversity criteria that fall into the *disparity* category of the diversity typology of [31] may have strong intrinsic interactions with criteria used for selecting a meritorious pool of candidates. As a simple example, if the number of publications is a criterion used during the initial merit selection stage, not accounting for the effect of career stage on publication record might inadvertently exclude junior participants. There may also be more subtle biases related to disparity-category diversity. If, for example, the application process requires a statement of motivation, and merit selection includes criteria based on this statement, this might subtly favour candidates who have had more experience or support in writing these statements, e.g. more senior candidates or those from better-resourced institutions. Because some of these disparity-category attributes, like privilege, access to travel funds and collaborations, may interact with demographic diversity in a number of systematic ways, selection outcomes in the first step might still be subtly biased against candidates from disadvantaged backgrounds even when the application procedure has removed names, addresses, and other proxies for disadvantage relevant to the specific cohort. This will be true even if merit selection criteria are based on well-designed rubrics, unless these interaction effects are taken into account. In these cases, unblinding the initial merit selection might engender fairer results than a blind procedure [94], but requires that committee members are sensitive to the present interactions, and how they might interplay with their own unconscious biases. It is worth noting in this context that this will be true for the second step as well: if, for example, programming experience is a category used during the second step, and target proportions are assigned such that they place heavy emphasis on previous experience with programming, this might disadvantage candidates who have had fewer opportunities to learn programming throughout the course of their careers.

Because results of the merit selection are passed on to the second, algorithmic stage, there is a strong likelihood that biases introduced in the first step will propagate through the second. Given the targets, *entrofy* may be able to correct biases introduced during the first step to a certain degree, but that is *not* its intended purpose. There are a number of biases associated with merit selection from the hiring literature, including gender, race and ethnicity [55, 58]. The hiring literature also provides some guidance towards building fairer procedures for merit selection. Proposed principles include the use well-designed rubrics across a number of merit categories for judging applicants in terms task-relevant skills; multiple, independent scores from a diverse set of evaluators for each candidate to avoid groupthink; quantitative evaluation to assess inter-rater reliability and unconscious biases [38]. We note that no matter how principled, the initial step of merit selection obscures part of the selection procedure in potentially significant ways and consequently detrimentally affects the desired transparency. We suggest that committees make as much of their procedure public as possible while protecting privacy of candidates, including application forms, rubrics and weights used for merit selection, and targets applied to *entrofy*.

It is up to the selection committee to determine how strongly to weight each step in the procedure. Our example workshop, Astro Hack Week, involved a minimal merit selection step and placed strong emphasis on the second, algorithmic part. We expect that many other real-world applications, where goals of the selection procedure strongly align with definitions of merit, will place a much stronger emphasis on the first step, and may use the second step only to minimally adjust outcomes. Categories where the targets are far away from the candidate set can be easily diagnosed from plots like shown in Fig 6, which can be generated by the *entrofy* software package. These visualizations should be generated for all steps in the procedure and all relevant categories of diversity to allow for evaluation of each step with respect to biases that may have been introduced. In cases where biases have been diagnosed, we suggest that organizers critically examine their initial recruitment and selection procedure for effects that could have resulted in generating a poorly representative set of meritorious candidates. For more practical advice for the application of *entrofy* in realistic problems, see also the S1 File.

It is the selection committee’s responsibility to define categories and targets that do not favour one group over another in ways that are misaligned with the goals of the selection. The definition of categories has a significant effect on the selection outcomes. This decision has to be made early, since information is generally elicited via an application form, and thus defining categories requires careful design before applications are solicited from candidates. In particular, care should be taken to calibrate categories that require self-assessment, such as the level of a particular skill or knowledge. When asking for self-assessment of skills, the available options should reflect measurable milestones (e.g. “I have written functions in Python before” when asking for programming skill) rather than broad, uncalibrated categories (e.g. “beginner” or “expert”). Overall, it is likely that this procedure is most useful to committees already sensitized to issues around diversity and committed to improving their procedures. In this case, objective gains made by employing *entrofy* as part of the overall strategy may be limited, but as discussed above, usage of the software and algorithm may free up the committee’s mental capacities for critically evaluating selection procedures.

The second, algorithmic step of the procedure is only of practical use if (1) the set of candidates is reasonably large, (2) the cohort to be selected is not very small, and (3) the difference between candidate set and selected cohort is not small (see also the results of our experiments, Fig 2). Together, cases (1) and (2) describe a problem that is common (e.g. in hiring), but subject to small-number statistics. Selecting a single employee out of a short-list of five to ten candidates is unlikely to benefit from algorithmic selection, and given well-defined rubrics a case where the committee may be able to hold all important information in memory. We note, however, that given the intrinsic variance of decision process in the employment context, one may consider generating a longlist or shortlist from a much larger pool of candidates using the two-step procedure above. Similarly, if the cohort to be selected is a very large fraction of the available meritorious candidates, then the space for affecting outcomes through algorithmically-mediated selection is very small. For example, if we aim to select a cohort of fifty out of a pool of fifty-five candidates, the selected cohort will likely closely resemble the overall make-up of the candidates, irrespective of the targets chosen. The precise limits on where this approach breaks down, however, are application-dependent.

As a matter of ethics, using a blind review followed by an unblinded algorithmic selection based on desired characteristics could have either a beneficial or *detrimental* outcome for individual participants and the cohort taken collectively. The target characteristics are determined by the selection committee, which leaves them subject to that group’s judgment. To the extent that *entrofy* is being used as envisioned, it is likely to lead to increased fairness for individual applicants around known biases and a more desirable cohort experience for those selected. However, in certain situations, blind review has been found to make selected groups *decrease* diversity [94]. This is especially the case for selection committees who already have a strong commitment to diversity and mechanisms in place to test for biases. In these cases, a blind first step may not be the optimal approach. We note that using demographic criteria like race, gender, and other identity statuses protected under employment law as targets in hiring decisions may require legal counsel beyond the scope of this software review.

As a final note, careful management of group diversity through a well-designed selection process can only ever be one part in a comprehensive strategy for building a group that is welcoming, inclusive and productive. While the diversity of a team has complex interactions with different outcomes, and adequate representation is important in itself, they need to mediated in practice by designs and processes that support attendees in engaging with the other participants. This includes instruments of social regulation like a Code of Conduct and associated enforcement strategies, active facilitation of activities and events that emphasize equitable participation (e.g. brainstorming [34], active learning strategies [95]), and feedback procedures that allow for in-the-moment responses to issues.

### Future work

While we have shown here that the algorithm correctly selects cohorts that closely adhere to the chosen targets where possible, the long-term effects of the proposed method on the resulting cohorts and its participants have not been systematically studied. In general, assessing the outcomes of a selection procedure is difficult for two reasons. First, defining “success” is non-trivial in many selection problems. Second, control groups almost never exist: we simply do not know how the second place job candidate would have performed, how successful a conference would have been if a different set of speakers had been selected or a different selection procedure was employed. Even clear evidence that conference attendance has a positive effect on early-career researchers is scarce.

In the future, the algorithm proposed here might open this line of inquiry in new ways. In particular, a crucial problem in studying conference outcomes is the selection problem: have participants been selected because they were already successful or has the conference improved their chances of success? The two-step procedure advocated here yields a pool of roughly equally qualified candidates some of whom were rejected (and did not attend) and others who were selected for attendance based on criteria besides merit (most of whom attended). Following the outcomes of individuals in both groups may provide a relatively clean data set for studying the effect conference/workshop/degree program attendance has on measures of success such as citations and career objectives. Anecdotally, we know from discussions and open-ended survey questions that participants favour diverse workshops. In particular, early-career researchers who identify as a gender and/or racial/ethnic minorities remarked positively on the representation of diverse demographic groups at Astro Hack Week. However, it is currently too early to assess the impact Astro Hack Week might have on the participants’ careers. How diversity interacts directly with hackathon outcomes is largely unexplored. An interesting direction of future research might bring together research on diversity in teams with recent work on hackathons to understand how diversity across different categories affects individual-level and group-level outcomes and what strategies are effective at mediating positive effects and mitigating negative effects at short, time-bounded participant-driven events.

Much of the discussion about the way *selection committees* engage with *entrofy* and how it changes committee members’ thinking about the procedure either draws on results from the hiring literature or is anecdotal. Similarly, we have not conclusively shown that a committee using *entrofy* makes objectively better decisions about a cohort than a committee performing a more traditional selection. In particular, it would be instructive to see a committee that traditionally has reported successes in selecting a diverse set of (unblinded) candidates adopt the procedure and compare the results. It would also be rewarding to study how the decision making process changes when committees utilize the approach suggested here, and if it indeed leads committees to ask critical questions about their selection, rephrase part of it in quantitative ways and use the data to improve future workshops.

Finally, cohort selection is a problem in many different contexts beyond participant selection for scientific workshops, which originally motivated this line of research. In the context of academic selections, the proposed framework can be extended to traditional conferences where talks, posters and papers are actively filtered. The similarities between this application and workshop participant selection invite a closely analogous procedure.

More generally, we envision that any cohort selection process could be a potential use case. In particular, we would like to explore applications of *entrofy* to undergraduate or graduate school admissions in which candidates are generally selected by committees similar in structure to conference selection committees. In this context, blinding during the first stage is difficult due to the nature of Curriculum Vitae and reference letters. Further, as discussed above, it may produce adverse results in situations where panels are particularly sensitive to diversity already. There is still merit, however, in making the process more quantitative. The overall applications could still be scored, and that scoring interrogated. As in the context of hiring, there is likely to be an large intrinsic variance in scores, and in general ranking decisions by committees may have low predictability especially when the decision process includes unstructured interviews, as e.g. a natural experiment at the University of Texas Medical School has shown [96]. Especially in highly competitive settings, which also includes, for example, grant proposals or fellowship applications, there is likely a large pool of highly qualified candidates and selection procedures might benefit considerably from accounting for committee variance in initial merit decisions by setting a fairly tolerant cut-off for acceptable candidates and letting *entrofy* decide between them based on other selection goals.

We do not anticipate *entrofy* to be useful for selecting employees in a typical hiring context, because it relies on maximizing a *cohort* over multiple categories, whereas many hiring decisions are usually performed for individual positions. However, *entrofy* could potentially be used in producing a balanced shortlist of candidates to be invited to interview or in cohort hiring scenarios as with first year law firm, banking, and consulting candidates [59] or seasonal cohort hiring.

## Conclusions

Cohort selection is and remains an intrinsically human, biased, and difficult problem. A plethora of evidence suggests that judgments based on the assessor’s experience and intuition often lead to selections that align with the assessor’s biases and lack predictive power with respect to selection outcomes. We suggest here that a two-step procedure based on a (potentially blind) merit selection followed by an algorithmic cohort selection based on extrinsic criteria can produce cohorts whose attributes align with target characteristics defined by the selection committee. We have presented a new algorithm and software, *entrofy*, that automates the second part of this process, have shown that it has found optimal solutions in practice, and have argued that this process provides accountability, transparency, and empowers humans to confront implicit biases in selection processes. The selected cohorts match pre-defined targets in both simulations and our case study, insofar as an optimal cohort exists. We propose that our solution allows for improved control of human biases during cohort selection, as well as greater accountability and fairness for candidates. However, research in academic cohort selection remains scarce and should be a priority for the future given the sustained effects of educational attainment on later life success metrics.

## Supporting information

S1 File Proof and practical advice. This file contains the proof that *entrofy* is NP-hard, as well as an example for how this procedure has been used in practice.
(PDF)
