# Supplementary material for: Entrofy your cohort: A transparent method for diverse cohort selection
Source: PLoS One. 2020 Jul 27;15(7):e0231939. doi: 10.1371/journal.pone.0231939 (PMC7384611; doi:10.1371/journal.pone.0231939)
Supplement: S1 File — This file contains the proof that entrofy is NP-hard, as well as an example for how this procedure has been used in practice. (PDF) [file pone.0231939.s001.pdf]

Supplementary Materials for: "Entrofy Your Cohort: A Data Science Tool to Maximize Diversity"

Daniela Huppenkothen,[1,2,3*] Brian McFee,[1] Laura Norén[1]

[1]Center for Data Science, New York University, 60 5th Avenue, 7th Floor, New York, NY 10003
[2]Center for Cosmology and Particle Physics, New York University
726 Broadway, 10th Floor, New York, NY 10003
[3]DIRAC Institute, Department of Astronomy, University of Washington,
3910 15th Ave NE, Seattle, WA 98195

*E-mail: dhuppenk@uw.edu

May 07, 2019

Entrofy is NP-Hard

The Entrofy optimization problem is closely related to the Hitting Set problem, which is known to be NP-Complete *(34)*.Here we formally prove that the Entrofy problem is also NP-Hard. We first restate the Entrofy problem in its decision form:

Problem 1 (Entrofy-decision)**.** *Given a set S of candidates, a collection of attributes $a_i : S \to \{0, 1\}$, a target size $k \in \mathbb{N}$, a set of target frequencies p_i for each attribute, does there exist a subset $X \subseteq S$ of size $|X| \leq k$ such that*

$$\forall i : \sum_{x \in X} a_i(x) \geq kp_i \, ? \tag{1}$$

To prove that problem 1 is NP-Hard, we reduce from the Hitting Set problem:

Problem 2 (Hitting set)**.** *Given a set S, a collection $C \subseteq 2^S$, and a positive integer k, does there exist a subset $X \subseteq S$ with $|X| \leq k$ such that for all $C_i \in C$, $|X \cap C_i| \geq 1$?*

Theorem 1. *The Entrofy decision problem (problem 1) is NP-Hard.*

Proof. Let (S, C, k) be an instance of Hitting Set. For each $C_i \in C$, let $a_i(x) = [x \in C_i]$, and let $p_i = 1/k$. Then $(S, \{a_i\}, k, \{p_i\})$ form an instance of the Entrofy problem. If this instance has a solution X, then that solution is also a solution to the Hitting Set problem, since

$$\sum_{x \in X} a_i(X) \geq kp_i = 1 \Leftrightarrow |X \cap C_i| \geq 1.$$

Similarly, if X is a solution to the Hitting Set problem, then it is also a solution to the corresponding Entrofy instance. □

Finally, we show that the optimization form of Entrofy is also NP-Hard.

Problem 3 (Entrofy-optimization). *Given inputs $(S, \{a_i\}, k, \{p_i\})$ as in problem 1, $\alpha > 0$, and weights $w_i \geq 0$, find a subset $X \subseteq S$ of size $|X| = k$ to maximize*

$$f(X) = \sum_i w_i \min\left(kp_i, \sum_{x \in X} a_i(x)\right)^{\alpha}. \tag{2}$$

Theorem 2. *The Entrofy optimization problem (problem 3) is NP-Hard.*

Proof. Again, we reduce from Hitting Set. Let (S, C, k) by an instance of problem 2. As before, let $p_i = 1/k$, a_i denote the indicator functions for C_i, and let $\alpha = 1$ and $w_i = 1$ for all i to form an instance of problem 3. Let $N = |C|$ denote the number of subsets, and let X be a maximizer of (2), which for this instance simplifies to

$$f(X) = \sum_i \min\left(1, \sum_{x \in X} a_i(x)\right) \leq N. \tag{3}$$

Note that each term of the sum is either 0 or 1, and indicates whether each C_i has a non-empty intersection with X. If $f(X) < N$, then some C_i is not covered by X. Since X maximizes (2), no better solution exists, so there cannot be a satisfying solution to the Hitting Set instance (S, C, k). Otherwise, $f(X) = N$, and X is a valid solution to the Hitting Set instance. □

Submodular optimization

Definition 1 (Submodular function). *A set-function $f : 2^S \to \mathbb{R}$ is* submodular *if for all $A \subseteq B \subset S$, and for all $x \in S \setminus B$,*

$$\Delta f(A, x) \geq \Delta f(B, x) \tag{4}$$

where Δ denotes the marginal gain*:*

$$\Delta g(T, y) := g\left(T \cup \{y\}\right) - g(T). \tag{5}$$

In plain language, submodular functions exhibit *diminishing returns*: the increase in f due to including a new element in the input set is non-increasing as the input set grows. Although efficient algorithms exist to minimize submodular functions, maximizing them is generally NP-Hard .If in addition to being submodular, the function is also monotone and non-negative, then a greedy maximization algorithm is guaranteed to find a solution X such that $f(X) \geq (1 - e^{-1})f^*$, where f^* is the optimal solution value.

Definition 2 (Monotone set-function)**.** *A set-function* $f : 2^S \to \mathbb{R}$ *is* monotone *if*

$$A \subseteq B \subseteq S \quad \Rightarrow \quad f(A) \leq f(B).$$

Note, however, the maximization of monotone submodular functions remains NP-Hard.

Submodularity of Entrofy

In this section, we prove that the Entrofy optimization objective (2) is monotone, non-negative, and submodular, and therefore admits an efficient greedy approximation algorithm. We first demonstrate that the individual attribute objectives

$$f_i(X) = \min\left(kp_i, \sum_{x \in X} a_i(x)\right)$$

satisfy these conditions, and then show that the non-negative weighted combination preserves these conditions. For ease of exposition, we will assume that kp_i is integral, though the results easily generalize to the non-integral case.

Lemma 1. *f_i is non-negative and monotone.*

Proof. Clearly, f_i is non-negative, since it is the minimum of two non-negative quantities. Let $A \subseteq B \subseteq S$. Then $\sum_{x \in A} a_i(x) \leq \sum_{x \in B} a_i(x)$ (because $a_i \geq 0$), so

$$\begin{aligned} f_i(A) &= \min\left(kp_i, \sum_{x \in A} a_i(x)\right) \\ &\leq \min\left(kp_i, \sum_{x \in B} a_i(x)\right) \\ &= f_i(B). \end{aligned}$$

□

Lemma 2. *f_i is submodular.*

Proof. Let $A \subseteq B \subset S$, and let $x \in S \setminus B$. This results in exactly one of three cases:

- If $f_i(A) < f_i(B) < kp_i$, then

$$\Delta f_i(A, x) = \Delta f_i(B, x) = a_i(x)$$

Neither A nor B saturates f_i, so including x changes both by $a_i(x)$.

- If $f_i(A) = f_i(B) = kp_i$, then

$$\Delta f_i(A, x) = \Delta f_i(B, x) = 0$$

Both A and B saturate f_i, so including x has no effect on either.

- If $f_i(A) < f_i(B) = kp_i$, then

$$\Delta f_i(A, x) = a_i(x) \geq \Delta f_i(B, x) = 0$$

B saturates f_i but A does not: including x increases the left-hand side by $a_i(x)$ and has no effect on the right-hand side.

In all three cases, the marginal gain condition (4) is satisfied, so f_i is submodular. □

Lemma 3. *For* $0 < \alpha \leq 1$, f_i^α *is non-negative, monotone, and submodular.*

Proof. Since f_i is non-negative, raising it a positive power α preserves non-negativity. Monotonicity and submodularity follows from the fact that submodular functions are closed under monotone concave transformations (**?**, *Theorem 1*). □

Theorem 3 (Entrofy submodularity)**.** *The Entrofy objective* $f(X) = \sum_i w_i f_i(X)^\alpha$ *is non-negative, monotone, and submodular.*

Proof. Lemmas 1 to 3 establish that the individual attribute objectives satisfy these conditions. Since these conditions are preserved by non-negative linear combination (**?**, *Section 2.1*), the total objective f is non-negative, monotone, and submodular. □

An Example Selection Procedure

In the following, we lay out in more detail the selection procedure for Astro Hack Week 2017 and give the reader a practical advice of how this selection can be performed.

Questions

Designing the application form is the most important and crucial step in the entire selection procedure, and the design itself should reflect the workshop type (e.g. a summer school might accept a different mix of researchers at different career stages than an unconference), the goals of the workshop itself and the organizers' values. In our experience as organizers, it is here that fruitful discussions about the workshop as a whole take place, and help shape not only the set of participants, but also the overall workshop agenda.

For Astro Hack Week, we include two categories of questions aside from personal data (e.g. name, e-mail address, institution): largely open-ended questions probing applicants' background and motivation, and multiple-choice questions about relevant demographic categories (e.g. gender and racial/ethnic background) as well as information specific to the workshop. For example, we asked participants about their subfield within astronomy, as well as to self-report their skill level in a range of methods relevant to Astro Hack Week, such as statistics, machine learning and programming.

Questions asking participants to self-report skill levels must be approached carefully, because terms like "beginner", "'intermediate" or "expert" have no absolute scaling and are thus subject to personal interpretation. Instead, we suggest calibrating skill levels on concrete milestones. For Astro Hack Week, we instead asked participants for example whether they had done machine learning in class work, used it in their research or had implemented their own methods. These questions should largely be phrased as multiple-choice questions (often with an option for candidates who do not fall into any of the categories). Answers to open-ended questions must be coded into numerical values before use in Entrofy, and might allow unintentional biases to enter the procedure.

Defining Target Distributions

For questions to be used within Entrofy, we recommend discussing the values for target distribution for each category *before* examining any of the applicants, unless Entrofy is used to reproduce input distribution for that category (which effectively amounts to a purely random selection in that category). Target distributions should be set according to the goals of the workshop, which are usually largely independent of the set of available applicants. It is important to note that these targets are entirely at the discretion of the selection committee, and in many cases, there likely is no "correct" answer. Targets overall should align with workshop goals and be transparent in the sense that the committee should be able to explain the reasoning behind them.

For example, at Astro Hack Week, we explicitly over-selected participants from gender and racial minorities compared to the overall population within astronomy, based on the reasoning

that one goal of the workshop was to increase representation of underrepresented groups within the technical fields of astronomy. Similarly, we set target fractions for the career stages of participants very deliberately: accepting 8% undergraduate participants allows very early-stage researchers to participate in the workshop and build valuable experiences and connections early on. This fraction was designed to be small enough not to dominate the workshop, but large enough (amounting to 4 out of 50 participants) for undergraduate participants to feel part of a representative group, rather than outliers. Targets for graduate students, postdoctoral fellows and faculty members (30%, 30% and 12%, respectively) were set to maximize exchange and learning between the different academic career stages. Finally, we accepted 10% research staff and 10% non-academic participants to allow for mixing between university and non-university staff as well as allow participants to build connection to industry. Similar reasoning was applied to all categories, aided significantly by earlier discussions about the application form and how the selection procedure relates to the overall goals of the workshop.

Initial Selection

We primarily used the responses to open-ended questions (blinded to personal characteristics) to understand applicants' motivations and decide whether they would be an overall good match for the workshop. At Astro Hack Week, we cast that net fairly wide, and only excluded duplicate applications as well as obvious spam responses (e.g. an Android developer aiming to develop a clearly astronomy-unrelated game for mobile phones). For other workshops that may be more narrowly defined, one approach could be to have every member of the organizing committee submit a score for each applicant (or, alternatively, a simple yes/no decision on each applicant), and then decide on a cut-off for acceptable candidates. It should be noted that this cut-off should not be too stringent, since committees should expect a large variance in individual decisions for a given applicant (**?**). At this stage, the committee may also examine scores for patterns indicating previously undetected biases and discuss cases with especially divergent scores. If the workshop or conference includes contributed talks or papers, they could be similarly included in this stage of decision making. Committees may also decide to vary their thresholds for acceptability in order to gauge the effects of that threshold on outcomes during the automatic stage of the selection.

Whether to blind this stage to applicants' demographic characteristics is a difficult decision. As we have discussed in the main text, unblinded scoring may lead to more severely biased decision making, while in some cases one may wish to include demographic characteristics as factors in the decision as a form of affirmative action. At Astro Hack Week, we chose to blind ourselves to the demographic characteristics of our applicants because the workshop was specifically aimed at a wide range of skill levels, and we therefore considered the risk of bias in an unblinded selection to be higher than excluding potential participants because of their background. However, we also aimed to remain vigilant about other sources of bias, for example in favour of native English speakers compared to non-native speakers.

It is possible to pre-select candidates during this stage. For Astro Hack Week, we automati-

cally pre-selected the organizing committee, invited tutorial teachers and several key participants. The latter were pre-selected after the initial selection stage, primarily to ensure the presence of experienced facilitators during Astro Hack Week. At Astro Hack Week, we limited the number of pre-selected participants to 20% in order to not bias the overall selection too much.

At this stage, we also surveyed the distributions of multiple choice questions to be included in Entrofy. In some cases, this required re-evaluation of the targets, especially in cases where some categories are not represented at all (e.g. for some of the larger categories with many options, like academic subfield) or very sparsely. In these cases, we have sometimes combined attributes to be slightly broader. Many multiple-choice questions also allowed for an "other" option in case applicants did not fall into any of the pre-defined categories. Evaluating these responses occasionally required creation of new attributes within certain categories and a subsequent re-evaluation of previously set targets.

Entrofy Selection

We used the results of the initial selection stage combined with the targets we defined to run Entrofy and select the remaining cohort of participants. Note that pre-selected participants were included as Entrofy as well. While they are automatically selected into the final set of participants, their attributes nevertheless count towards the targets we set. If we had pre-selected participants who are overrepresentative of the targets in one or several categories, the algorithm automatically tries to balance this overselection.

Evaluation

There are different ways in which organizers may wish to evaluate the validity and robustness of their selection. As shown in Figure (4), we found figures comparing the input set, the targets and the output set particularly helpful. These plots allowed us to quickly diagnose how closely the selected cohort matched the target, and check for categories with a large mismatch between the pool of applicants and our envisioned targets. Because the initial stage of selection was fairly unrestrictive, we have not performed the same exercise between the overall pool of candidates and those selected during the first step. In more restrictive selection problems, doing so might help diagnose where candidates with certain attributes are preferentially selected or rejected initially, and thus help in controlling for bias during the first stage.

During the Astro Hack Week selection, we have also included figures that plot representation in both the full pool of candidates as well as the selected cohort for combinations of different categories. This was particularly helpful to diagnose strong correlations in our pool of applicants: for example, we wanted participants identifying as gender or racial minorities to be represented at all career stage, such that junior participants would have a diverse set of role models available. If this had not been the case, we could have re-evaluated these categories and combined them into a new category that includes combinations of attributes relating to gender, race/ethnicity and career stage.

As mentioned in the main text, these diagnostics have been helpful beyond the selection procedure. In particular, they have helped us with targeting outreach initiatives ahead of subsequent workshops, made us conscious of recruiting a diverse set of tutorial teachers, and have been used in funding applications aimed at boosting the attendance of junior participants, particularly from underrepresented minorities.

Transparency

One of the primary goals of this procedure is to provide transparency about the decision making process to participants, but explicitly also to those that were rejected. In the context of the workshops that have used Entrofy as part of their selection, we have experimented with different formats of reporting. At all workshops, we communicated the basics of the selection procedure at the application stage via the application form. At the workshop itself, we provided more details about the selection procedure, usually on the first day. For Astro Hack Week, we have in the past documented and published the procedure as part of a series of blog posts. For another workshop, we have also published records of the procedure in the form of a Jupyter notebook including a randomized simulated data set based on the original input set (to preserve privacy) and the original code used to perform the Entrofy selection.
